# Supplementary material for: Predicting Affinity Through Homology (PATH): Interpretable binding affinity prediction with persistent homology
Source: PLoS Comput Biol. 2025 Jun 27;21(6):e1013216. doi: 10.1371/journal.pcbi.1013216 (PMC12226026; doi:10.1371/journal.pcbi.1013216)
Supplement: S1 Text — Sect A details the precise definition of persistent homology and the construction of IPCs, which are the inputs to the regression trees of PATH+ and PATH−. Sect B details the process by which we curated the features used to construct persistence fingerprint from persistence images, and justifies our choice of hyperparameters, such as the number of features and the number of regression trees. Sect C explains the high complexity that is obtained by naively computing IPCs, and the high complexity of previous binding affinity prediction algorithms based on persistent homology (Sect C.1). It also elucidates a fast and provably ε-accurate approximation for persistence fingerprint, which (without our fast approximation algorithm) would naively have inherited the high computational complexity of IPCs. Sect D shows the decision trees of PATH+. Sect E shows the results of benchmarking PATH+ and PATH− against previous binding affinity prediction algorithms in numerical tabular form, to complement the plots made in main MS Fig 1. Sect F lists the top 77 features selected by the highest mean decrease in impurity in the 120 initial persistence images constructed in Sect B.1. (PDF) [file pcbi.1013216.s001.pdf]

Supplementary Information (S1 Text)

Predicting Affinity Through Homology (PATH): Interpretable Binding Affinity Prediction with Persistent Homology

Yuxi Long[1], Bruce R. Donald[1,2*]

[1]Department of Computer Science, Department of Mathematics. Duke University, Durham, North Carolina, United States of America

[2]Department of Biochemistry, Department of Chemistry. Duke University and Duke University School of Medicine, Durham, North Carolina, United States of America

*Corresponding author: Bruce R. Donald. Email: `brd+pcb25@cs.duke.edu`. This research was supported by NIH grant R35 GM-144042 to B.R.D.

This supplementary information provides additional information to substantiate the claims made in the main paper.

In this appendix, all distances are expressed in Angstroms; hence, the unit is omitted in the text. All logarithms are natural logarithms. To avoid confusion with vector norms, which are denoted by $||\cdot||$, the cardinality of a set X is denoted by $\overline{\overline{X}} = |X|$. The figures in this appendix are numbered differently from the figures in the main paper. Intermediate lemmas toward Theorem 1 and their proofs are nested within the proof of this theorem. For the sake of clarity, proofs of lemmas are ended with a $\square$ symbol, while proofs of theorems are ended with a $\blacksquare$ symbol.

Section A details the precise definition of persistent homology and the construction of IPCs, which are the inputs to the regression trees of PATH$^+$ and PATH$^-$. In particular, Section A.1 formally defines the construction of persistent homology and persistence diagrams; Section A.2 formally defines the construction of persistence images, which is similar to IPCs; And finally, Section A.3 formally defines the construction of IPCs.

Section B details the process in which we curated the features used to construct persistence fingerprint from persistence images and justifies our choice of hyperparameters, such as the number of features and the number of regression trees. In particular, Section B.1

describes the process of selecting important features measured by their mean decrease in purity; Section B.2 describes selecting the most important features among the features resulting from Section B.1 using an *ablate-and-test* procedure; Section B.3 shows the intermediate results of the previous two subsections and supports our choice of 10 persistence fingerprint components and 13 regression trees in $PATH^+$; Finally, Section B.4 confirms that decision trees are still the optimal choice of model, given these 10 selected features as input. Finding that the top 10 important features all belong to persistence images from the persistence of 0D and 1D homology groups constructed using the opposition distance (d_{op}), we interpreted their geometric relevance and developed a one-dimensional analog of persistence images, which we called internuclear persistence contours (IPCs) (Main MS Section 4.3). Such a one-dimensional representation is only possible with the persistent homology of 0D and 1D Vietoris-Rips filtration constructed with the opposition distance, where all of the persistence fingerprint components come from. Section B.5 shows the generalizability of persistence fingerprints using boxplots and PaCMAP [52].

Section C explains the high complexity that is obtained by naively computing IPCs and the high complexity of previous binding affinity prediction algorithms based on persistent homology (Section C.1) and elucidates a fast and provably ε-accurate approximation for persistence fingerprint, which would naively have inherited the computational complexity of IPCs. Derivation of the asymptotic complexity of this approximation and proof of its accuracy are done in Section C.2 and Section C.3. Experimental runtime and accuracy for this approximation is reported in Section C.4 and validate the complexity claims and proofs in Sections C.2 and C.3.

Section D shows the decision trees of $PATH^+$. In particular, Section D.1 explains how to interpret each decision tree; Section D.2 shows the full set of decision trees.

Section E shows the results of benchmarking $PATH^+$ and $PATH^-$ against previous binding affinity prediction algorithms in numerical tabular form, to complement the plots made in main MS Fig 4.

Section F lists the top 77 features selected by the highest mean decrease in impurity in the 120 initial persistence images constructed in Section B.1.

A Definition of Persistent Homology

A.1 Construction of Persistent Homology

We describe persistent homology using notation from [119, 29, 120, 121]. Chapter 50 of [31] gives an in-depth summary of computational topology on protein structure. Visualizations of growing filtration parameters in persistent homology are made using a Mathematica notebook by Adams and Sergert [122]. Computation of persistent homology and persistence images are done using GUDHI [123], giotto-tda [124], giotto-ph [125], and Ripser [126].

A.1.1 Simplicial Homology.

A *simplex* is a generalization of a triangle to arbitrary dimensions. An n-simplex is the convex hull of $n + 1$ points and is denoted by a square bracket around its vertices. As an example of this notation, $[v_0, v_1, v_2, ..., v_n]$ denotes an n-simplex made up of the vertices $\{v_0, v_1, ..., v_n\}$. Note that the order of the vertices in a simplex is important. The *face* of a simplex is a simplex with vertices any nonempty subset of the v_i's. For example, the two-dimensional faces of a 3-simplex (a solid tetrahedron) are four 2-simplices (filled triangles), and the one-dimensional faces of a 2-simplex (a filled triangle) are three 1-simplices (line segments).

A finite *simplicial complex* K is a finite collection of simplices such that for any three simplices σ, τ, σ_0

1. If $\sigma \in K$ and $\tau \leq \sigma$, then $\tau \in K$.

2. If $\sigma, \sigma_0 \in K$, then $\sigma \cap \sigma_0$ is either empty or a face of both.

In this manuscript, we assume finite simplicial complexes when referring to simplicial complexes. Given a simplicial complex K, we can define $C_n(K)$ as the vector space generated by the n-simplices in K as basis over a ring. Herein, the ring is chosen to be $\mathbb{Z}$. Define an *n-chain* to be the formal sum $c = \sum_j \gamma_j \sigma_j$, where $\gamma_j \in \mathbb{Z}$ are the ring elements and σ_j are the n-simplices in K. Then $C_n(K)$ consists of all the n-chains.

We define the *boundary operator* ∂_n on a n-simplex to be an alternating sum of its $(n - 1)$ dimensional faces. Formally, $\partial_n([v_0, v_1, ..., v_n]) = \sum_{i=0}^{p} (-1)^i [v_0, v_1, ..., \hat{v}_i, ..., v_n]$, where the hat in $\hat{v}_i$ means that $[v_0, v_1, ..., \hat{v}_i, ..., v_n]$ is the simplex formed by removing the i-th vertex in $[v_0, v_1, ..., v_n]$. For example, $\partial_1([v_0, v_1]) = [v_1] - [v_0]$ and $\partial_2([v_0, v_1, v_2]) = [v_1, v_2] - [v_0, v_2] + [v_0, v_1]$. The boundary of an n-chain is obtained by extending ∂_n linearly, $\partial_n(c) = \sum_j \gamma_j \partial(\sigma_j)$, where σ_j are the n-simplices in c. Then ∂_n defined on the n-chains

is a homomorphism: $C_n(K) \to C_{n-1}(K)$. This gives us a sequence of chains connected by boundary homomorphisms, called *chain complexes*:

$$\ldots \xrightarrow{\partial_{n+2}} C_{n+1} \xrightarrow{\partial_{n+1}} C_n \xrightarrow{\partial_n} C_{n-1} \xrightarrow{\partial_{n-1}} \ldots$$

Note that $\partial_{n-1} \circ \partial_n(c) = 0$ for any integer n and any $c \in C_n(K)$. The n-chains that have boundary 0 (i.e., in the kernel of ∂_n) are called *n-cycles* and denoted $Z_n(K)$. The n-chains that are the boundary of $(n+1)$-chains (i.e., in the image of ∂_{n+1}) are called *n-boundaries* and denoted $B_n(K)$. The *n^{th} homology group* $H_k(K)$ is the quotient group $Z_n(K)/B_n(K) = \ker(\partial_n)/\mathrm{im}(\partial_{n+1})$. The fact that $\partial_n \circ \partial_{n+1}(c) = 0$ tells us that $B_n(K) \subset Z_n(K)$. Note that $B_n(K)$ is a normal subgroup because $Z_n(K)$ is abelian, so $H_n(K)$ is well-defined. By the structure theorem for finitely generated abelian groups, $H_n(K) \cong \mathbb{Z}^r \oplus (\mathbb{Z}/d_1\mathbb{Z}) \oplus (\mathbb{Z}/d_2\mathbb{Z}) \oplus \ldots \oplus (\mathbb{Z}/d_m\mathbb{Z})$, where $d_1|d_2|\ldots|d_m$ and the symbol $\cong$ denotes group isomorphism. Respectively, $\mathbb{Z}^r$ and $\bigoplus_{i=1}^{m}(\mathbb{Z}/d_i\mathbb{Z})$ are called the *free part* and the *torsion part* of this homology group [31]. Geometric objects that are embeddable in $\mathbb{R}^3$ such as the simplicial complex for a protein-ligand complex will generally have zero torsion [31].

The *n^{th} Betti number* of $H_n(K)$, denoted β_n, is equal to r, the rank of the free part of $H_n(K)$. Geometrically, the nth Betti number can be understood as the number of n-dimensional holes in the complex [120]. These terms will be used interchangebly for this manuscript.

A.1.2 Persistence.

Given a simplicial complex K, a *filtration* of K is a sequence of subcomplexes such that

$$\varnothing = K_0 \subset K_1 \subset \cdots \subset K_n = K.$$

Given a set X with a distance function $d : X \times X \to \mathbb{R} \cup \{+\infty, -\infty\}$, we can construct the *Vietoris-Rips complex*[1] *of X with parameter* $r \in [0, \infty)$, denoted $\mathbf{VR}_r(X)$, by forming a simplex for every finite set of points in which every pair of points has distance at most r. The Vietoris-Rips filtration of X is then defined as $\{\mathbf{VR}_r(X)\}_{r\in\mathbb{R}}$. Note that for any $r \leq s$, we have $\mathbf{VR}_r(X) \subset \mathbf{VR}_s(X)$. Therefore, the Vietoris-Rips filtration is a filtration of $\mathbf{VR}_\infty(X)$.

The Vietoris-Rips filtration is indexed by $\mathbb{R}$, an uncountably infinite set. However, if X is

[1] Edelsbrunner and Harer [29] define the Vietoris-Rips complex with filtration parameter r as $\mathbf{VR}_r(S) = \{\sigma \subset S : \text{diam } \sigma \leq 2r\}$. We use the definition from Zomorodian and Carlsson [106] as it is consistent with our implementation. These two alternative definitions for the Vietoris-Rips complex can be reconciled simply by a rescaling of the filtration parameter.

finite, then $\mathbf{VR}_r(X)$ can be represented by a filtration with a finite number of subcomplexes without losing information [126]. Intuitively, the information contained in $\{\mathbf{VR}_r(X)\}_{r\in\mathbb{R}}$ can be entirely captured by subcomplexes before and after the formation of each simplex, and there is a finite number of simplices in $\mathbf{VR}_\infty(X)$ for finite X.

While the Vietoris-Rips filtration is usually defined for metric functions, it can be easily seen that the Vietoris-Rips filtration is well-defined for the opposition distance (Eq. 2 in the Main MS) as well.

A *homology class* is an element of a homology group, termed a "class" because the group is expressed as a quotient. A homology class α is defined to be *born* at K_i if it is not in the image of the map induced by the inclusion $K_{i-1} \hookrightarrow K_i$. Furthermore, if α is born at K_i, then it *dies entering* K_j if the image of the map induced by $K_{i-1} \hookrightarrow K_{j-1}$ does not contain the image of α but the image of the map induced by $K_{i-1} \hookrightarrow K_j$ does.

We can precisely represent the birth and death of homology classes of a certain dimension using a *persistence diagram*, where each point (x, y) corresponds to an n-dimensional homology class (an n-dimensional hole) that appears (is born) at scale x and disappears (dies) at scale y. We denote the nth persistence diagram as $D_n(X)$ and define $D(X) = \{D_n(X)\}_{n\in\mathbb{Z}_{\geq 0}}$. Persistence diagrams has been shown to be stable with respect to small perturbations in input:

Theorem 2 (Stability of persistence diagrams [127, 128])**.** *For finite metric spaces (X, d_X) and (Y, d_Y), we have $d_b(D(X), D(Y)) \leq 2d_{GH}(X, Y)$. Here d_b and d_{GH} are the bottleneck distance and the Gromov-Hausdorff distance respectively between persistence diagrams and metric spaces [127, 128].*

Proof of Theorem 2 can be found in [127, 128].

Thus, in embedding biochemical structure, small differences in the protein structure which arise often due to the structural heterogeneity of proteins [42] will have little effect on the resulting representation.

A.2 Persistence Images

The concept of *persistence image* was developed by Adams et al. [108] as a stable vectorization method for persistence diagrams. Let $T : \mathbb{R}^2 \to \mathbb{R}^2$ be the linear transformation $T(x, y) = (x, y - x)$. Let $\phi_u : \mathbb{R}^2 \to \mathbb{R}$ be a differentiable probability distribution with mean $u \in \mathbb{R}^2$. Additionally, fix a nonnegative weighting function $f : \mathbb{R}^2 \to \mathbb{R}$ that is zero along the horizontal axis, continuous, and piecewise differentiable. Then the *persistence surface* of a persistence diagram $D_n(X)$ is defined as the continuous function $\rho_{D_n} : \mathbb{R}^2 \to \mathbb{R}^+$ given by

$$\rho_{D_n}(x,y) = \sum_{u \in T(D_n)} f(u)\phi_u(x,y). \quad (3)$$

Hence, the persistence surface of a diagram is the sum of translated and weighted Gaussian kernels centered at persistence of each homology class, represented by its birth and death radii as coordinates (x, y) in the 2D plane. Given a positive pixel width $w \in \mathbb{R}^+$, we can construct a partition of $\mathbb{R}^2$ into square *pixels* with side length w, which we denote by $I_{Q_w}(\rho_{D_n(X)})$ is the collection of pixels $I_{Q_w}(\rho_{D_n(X)}) = \{\iint_q \rho_{D_n(X)} dydx : q \in Q_w\}$.

Theorem 3 (Stability of persistence images [108])**.** *The persistence image with Gaussian distributions is stable with respect to the 1-Wasserstein distance between diagrams.*

Proof of Theorem 3 can be found in [108].

Persistence images are provably stable with respect to persistence diagrams, and thus stable with respect to perturbations by Theorem 2.

While the persistence image is defined over all of $\mathbb{R}^2$, in this manuscript, only the subset of pixels in the range $[0, 50] \times [0, 50]$ (in Angstroms) are used. In this manuscript, each persistence image is created with standard deviation of Gaussian kernel $\sigma = 0.1$, and the width of each pixel is 0.5 Å. The persistence images are then flattened into a 10,000-dimensional vector as inputs to regression algorithms (Section B). PATH$^+$ and PATH$^-$ uses persistence images from dimensions $n = \{0, 1\}$, and TNet-BP uses persistence images from dimensions $n = \{0, 1, 2\}$.

A.3 Definition of Internuclear Persistence Contours (IPCs)

As discussed in the beginning of Section 4.3, the persistence of each 0D or 1D homology group constructed with the opposition distance can be captured with a single scalar value (death radius for 0D, birth radius for 1D). We call each of these scalar values a *critical value* [31] of the filtration. Then the 0D or 1D persistent homology information can be captured by a sequence of critical values $\{r_1, r_2, ..., r_n\}$ without losing information. In the following subsection, we formalize this intuition.

Fix a protein-ligand complex and fix a persistence fingerprint component. Fixing the persistence fingerprint component fixes a subset of protein atoms P and a subset of ligand atoms L matching certain element types[2]. The protein atom subset P, ligand atom subset L

[2]Fixing the persistence fingerprint component also fixes a bin I and a dimension (0 or 1) such that this persistence fingerprint component is the integral over I of the IPC in that dimension (0 or 1) constructed with the atoms P and L. This information is not needed for computing IPCs, but will be useful to us when calculating the persistence fingerprint.

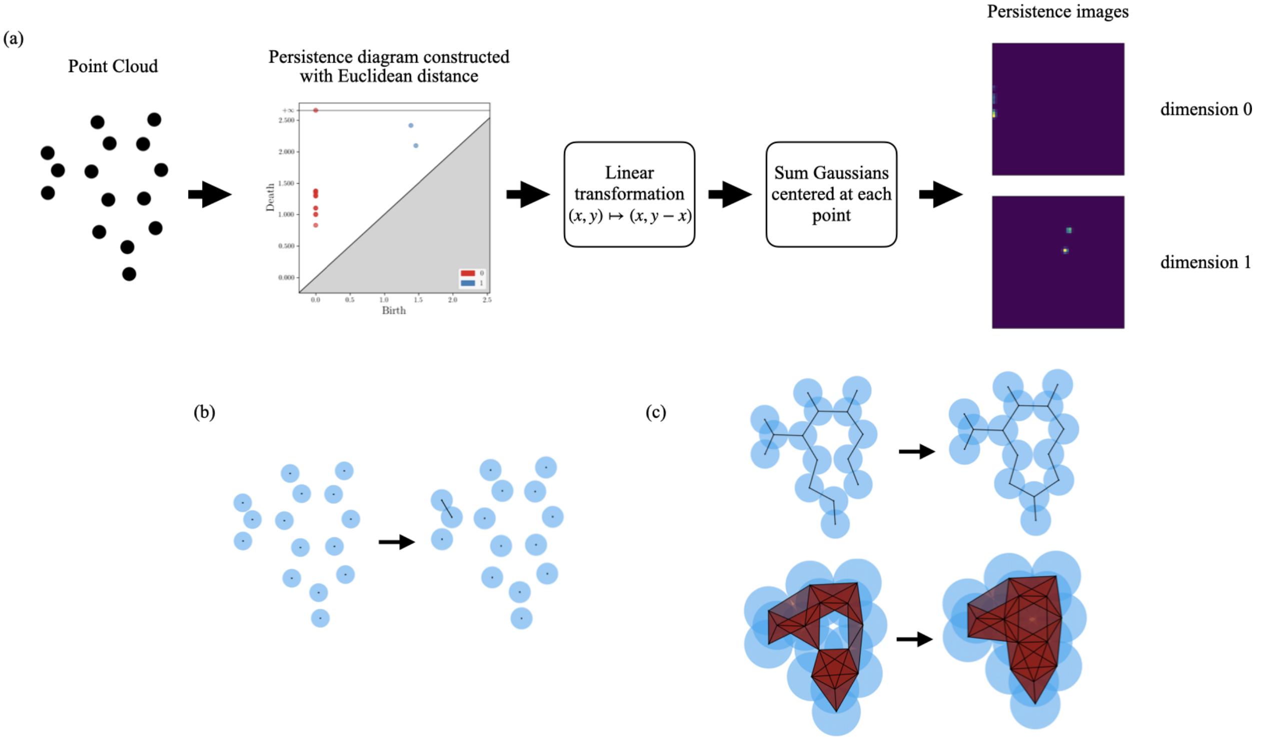

Figure A: **Construction of persistence images with the Euclidean distance.** **(a)** Example construction of a persistence image using an adenine molecule and Euclidean distance as an example. Note two differences between this illustration and construction of persistence fingerprints (detailed in Section A.2). One, while this figure only shows persistence image constructed with the Euclidean distance, persistence fingerprint includes features constructed with d_{op}, which is a different distance function (Table 1).Two, the adenine molecule is planar, hence can be shown in 2D, but the actual construction of persistence fingerprints are done in 3D. Details are left to Section A.2 **(b)** Disappearance of a connected component (i.e., zero-dimensional hole) at $r = 0.8$. Note that the disconnected components had existed since $r = 0$. These correspond to the point on the persistence diagram with coordinates (0, 0.8). **(c)** Appearance of a one-dimensional hole at $r = 1.4$ and disappearance at $r = 2.5$. This corresponds to the point on the persistence diagram with coordinates (1.4, 2.5).

for each persistence fingerprint component are given by Table 1 in the Main MS. For example, if we fix the 5th persistence fingerprint component, we read from Table 1 the information

**Protein Atom**	**Ligand Atom**	**IPC Dimension**	**IPC Density Bin (Å)**
N	C	1	[10.0, 10.5]

then P is the subset of all protein nitrogen atoms, L is the subset of all ligand carbon atoms[3].

[3]We also learn that for this component, $I = [10.0, 10.5]$, but this information is only needed for construction of persistence fingerprint and will not be needed for construction of IPC.

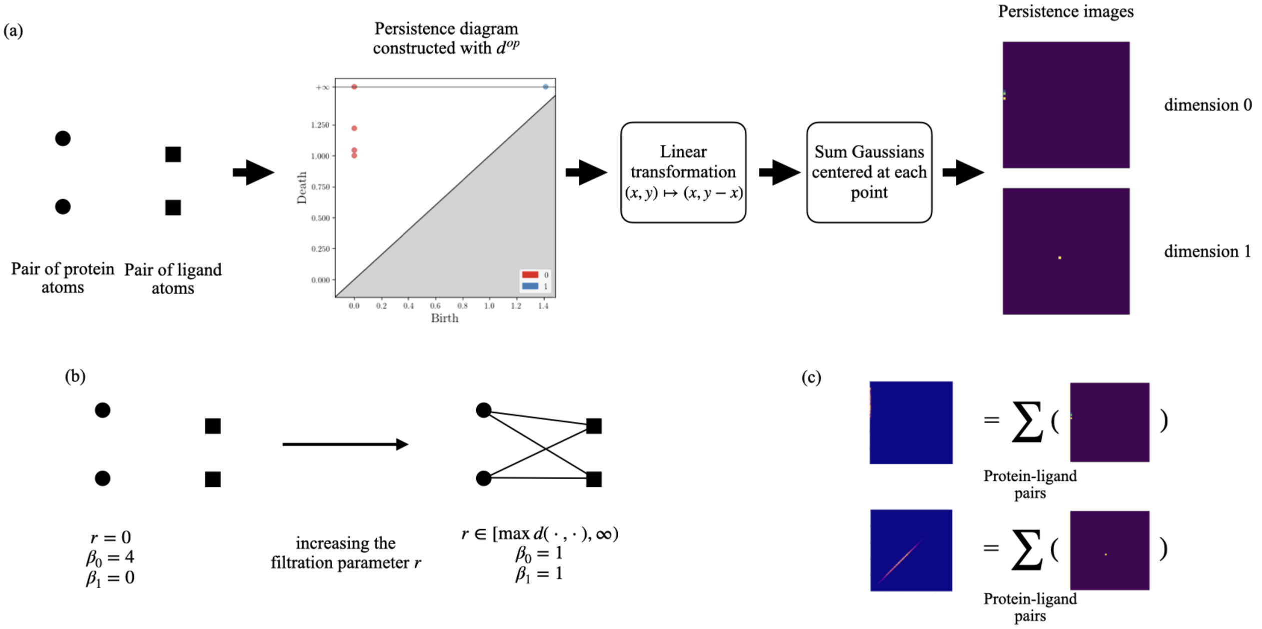

Figure B: **Construction of persistence images with d_{op}.** **(a)** In the persistence diagram, a pair of protein atom and a pair of ligand atoms will create 4 points representing 0-dimensional holes, and 1 points represent a 1-dimensional hole. This results in a few bright areas on the left edge of the persistence image for dimension 0 and a bright spot on the diagonal of the persistence image for dimension 1. **(b)** Illustration of how d_{op} creates a 1-dimensional hole in the persistence diagram representation for the two pairs of atoms: For protein atoms p_1, p_2 and ligand atoms l_1, l_2, when the filtration parameter r increases beyond $\max\{d(p_i, l_j) : i, j \in \{1, 2\}\}$ distance among the atoms, there is always a 1-dimensional hole (Lemma 1). Intuitively (see Lemma for proof), there never exists a filled triangle because the distance between two atoms with the same affiliation (protein or ligand) is ∞. **(c)** The persistence image representation of a protein-ligand complex is the sum of the individual interactions at different scales, taking multiplicity into consideration.

Then the protein-ligand complex, which we notate as $S = P \sqcup L$, is a disjoint union of the atoms in P and L, while each atom additionally has information of its affiliation (whether it came from the protein or the ligand). The filtration in concern in the remaining proof will be the Vietoris-Rips filtration of S of filtration radius r with d_{op}, which is $\mathbf{VR}_r(S)$.

Lemma 1 (Persistence of 1D holes). *A hole in the 1D Vietoris-Rips filtration with d_{op} as distance function is generated by a 1-cycle and never dies. The number of 1D holes in this construction monotonically increases with increasing filtration radius r.*

Proof of Lemma 1: Because $B_2 = \text{im}(\partial_2)$ is generated by the faces of 2-simplices, each element in its basis must be of the form $[v_y, v_z] - [v_x, v_z] + [v_x, v_y] = \partial([v_x, v_y, v_z])$ resulting from some 2-simplex $[v_x, v_y, v_z]$. However, for any three atoms $\{x, y, z\} \in S$, two of them must have the same affiliation (either P or L), and the opposition distance between these two atoms with the same affiliation (i.e., both atoms are from the protein or both atoms are from the ligand) is ∞. This means that $[y, z] - [x, z] + [x, y]$ cannot be a generator for B_2. Therefore, for any filtration radius r, we have $B_2 \cong 0$, where the symbol $\cong$ denotes group isomorphism.

Then for any positive r, we have $H_1 \cong \ker(\partial_1) = Z_1$, which are the 1-cycles. Obviously, no 1-cycle perishes with increasing r. As a result, the rank of the 1st homology group (i.e., number of 1D holes) monotonically increases with increasing filtration radius r. $\square$

Lemma 2 (Critical values of filtration with d_{op}). *The persistence of each 0D and 1D hole in the Vietoris-Rips complex constructed with d_{op} can be faithfully characterized by a single scalar value. Namely, death radius for a 0D hole and birth radius for a 1D hole. Name this scalar value the* critical value *of the hole, then the set of critical values R for a filtration satisfy*

$$R \subset \begin{cases} \{d(p, l) : p \in P, l \in L\} & \textit{for 0D} \\ \{\max_{e \in E} w(e) : m = (V, E) \in M\} & \textit{for 1D} \end{cases} \tag{4}$$

Where M is defined as the set of all cycles m generated by a sequence of alternating protein atoms and ligand atoms, and each cycle is viewed as a graph in which each edge e has a weight $w(e)$ equal to the Euclidean distance between the two atoms it connects.

Proof of Lemma 2: An intuition of this proof can be found in Fig B. Because the rank of the 0th homology group H_0 of a simplicial complex is just number of connected components in this complex, each 0D hole is born at $r = 0$. Hence the persistence of each 0D hole can be

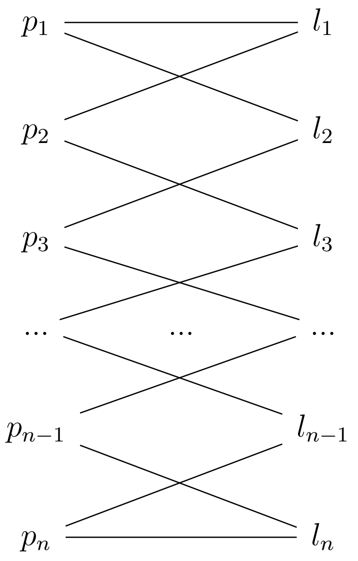

Figure C: Example of a 1-cycle in the Vietoris-Rips filtration of a protein-ligand complex constructed with opposition distance. Notice that this 1-cycle is generated by a sequence of alternating protein atoms and ligand atoms.

characterized only by the death radius of the hole, which is distance between a protein and a ligand atom, which is in the set of all distances between protein and ligand atoms. Thus the 0D case in Lemma 2 holds.

For 1D holes, the death radius is ∞ by Lemma 1, so the persistence of each 1D hole can be characterized only by the birth radius of the hole, which is the birth radius of a 1-cycle by Lemma 1. Note that because $d_{op}(p_i, p_j) = \infty$ and $d_{op}(l_i, l_j) = \infty$, a 1-cycle in d_{op} must consists of a sequence of alternating protein atoms and ligand atoms and looks like $Z = [p_1, l_1] + [l_1, p_2] + [p_2, l_3] + [l_3, p_4] + ... + [l_{n-1}, p_n] + [p_n, l_n] + [l_n, p_{n-1}] + [p_{n-1}, l_{n-2}] + ... + [p_3, l_2] + [l_2, p_1]$ (Fig C). Let the elements in this sum be labeled $\zeta = \{(p_i, l_j) : [p_i, l_j] \in Z \vee [l_j, p_i] \in Z\}$. Then this 1-cycle has birth time $\max_{(p_i,l_j)\in\zeta} d(p_i, l_j)$.

Z can be viewed as a weighted graph $m = (V, E)$ with vertices $V = \{p_i\} \cup \{l_i\}$, edges $E = \zeta$ and let each edge be endowed the Euclidean distance between its vertices as its weight. Since the critical value of every hole in H_1 is generated by such a graph, the 1D case in Lemma 2 holds. Note that m is similar to a bipartite matching between the protein and ligand atoms (Fig C), hence we refer to the construction of 1D homologies as *bipartite matchings of the protein and ligand atoms.* □

With Lemma 2, we know that the persistent homology constructed on a protein-ligand complex in dimensions 0 and 1 can be faithfully represented by a sequence of critical values R. Thus, we can define the *Internuclear Persistence Contour (IPC)*, which is a non-negative

real valued function $\gamma : \mathbb{R} \to [0, \infty)$. To construct the (continuous) IPC, we sum Gaussian kernels centered at each of the critical values (r_i's). Let us denote a Gaussian kernel with mean μ and standard deviation σ by $g_\mu(x)$. The IPC is defined as follows:

$$\gamma(x) = \sum_{i=1}^{n} g_{r_i}(x). \tag{5}$$

Note the similarity of γ with the definition of persistence image ρ (from Section A.2), because γ is a special case of ρ for Vietoris-Rips filtration constructed with the opposition distance d_{op} (Section 4.2) in dimensions 0 and 1, which only has one degree of freedom for the persistence of each homology group.

In our current implementation, the zero-dimensional IPCs are approximated by taking the left-most column of pixels of the 0D persistence image and the one-dimensional IPCs are approximated by taking the diagonal of pixels of the 1D persistence image. Hence, in PATH, g_{r_i} is defined as follows:

$$g_{r_i}(x) = \int_0^{0.5} \frac{1}{2\pi\sigma^2} \exp\left(-\frac{(x - r_i)^2 + y^2}{2\sigma^2}\right) \mathrm{d}y, \tag{6}$$

where x and y can be regarded as coordinates of the horizontal and vertical axes, respectively, of the two dimensional persistence image. This can be easily computed by existing packages for topological data analysis [129, 124], and behaves sufficiently similar to the Gaussian kernel so that the computational complexity and approximation error guarantees (Section C) still hold. In a future implementation, we plan to use the exact Gaussian kernel instead of this approximation.

Given a point cloud, we can compute the 0D and 1D persistent homologies with d_{op} and construct two IPCs from it: we call IPCs constructed using 0D death times the *0D IPC*, and IPCs constructed using 1D birth times the *1D IPC*. Definition of *IPC density* follows by integrating its underlying IPC over equally spaced bins. For this IPCs in this paper, the bins are constructed with a width of 0.5 starting from 0 (i.e., bins $= \{[0, 0.5], [0.5, 1.0], [1.0, 1.5]...\}$), and the IPC densities of a certain IPC γ will be the ordered set $\left\{\int\limits_I \gamma(x)\mathrm{d}x : I \in \text{bins}\right\} = \left\{\int_0^{0.5} \gamma(x)\mathrm{d}x, \int_{0.5}^{1.0} \gamma(x)\mathrm{d}x, \int_{1.0}^{1.5} \gamma(x)\mathrm{d}x, ...\right\}$. IPC density provides a fixed-size vectorization of IPCs. Stability of IPC density follows from the stability of persistence images (Theorem 3).

Table A: **Atom subsets and distance functions used to construct persistent homology for the *initial images*.** The set of protein atoms is denoted P and the set of ligand atoms is denoted L. The set of heavy atom types in the protein is defined as $P_e = \{\text{C, N, O, S}\}$, and the set of heavy atom types in the protein is defined as $L_e = \{\text{C, N, O, S, P, F, Cl, Br, I}\}$. These are exactly the atom subsets and distance functions used to construct the features of TNet-BP [43]. We construct a persistence image for each of the homology dimensions $\{0, 1, 2\}$ in each of these atom subsets.

Set	Atoms Used	Distance Function
0-35	$\{a \in P : T(a) = e_{\text{P}}\} \cup \{a \in L : T(a) = e_{\text{L}}\} : e_{\text{P}} \in P_{\text{e}}, e_{\text{L}} \in L_{\text{e}}$	d_{op}
36	$\{a \in P : T(a) \in P_{\text{e}}\}$	Euclidean
37	$\{a \in P : T(a) \in P_{\text{e}}\} \cup \{a \in L : T(a) \in L_{\text{e}}\}$	Euclidean
38	$\{a \in P : T(a) = \text{C}\}$	Euclidean
39	$\{a \in P : T(a) = \text{C}\} \cup \{a \in L : T(a) = \text{C}\}$	Euclidean

B Feature Selection

B.1 Feature Selection by Mean Decrease in Impurity (MDI)

B.1.1 Initial images.

This section describes the process in which we selected the features used to construct persistence fingerprint from IPCs.

We created 40 subsets of atoms of from the protein and ligand (Table A). From each subset of atoms, a persistence diagram is constructed for each of the homology dimensions $\{0, 1, 2\}$. Subsequently, three persistence images are created, one for each dimension. The resulting 120 persistence images constitute a superset of the features constructed in TNet-BP [43]. We call this set of persistence images the *initial images*.

B.1.2 Finding the important features for prediction.

We trained gradient boosting regressors (GBRs) [130] using the set of flattened initial images of each complex the PDBBind v2020 refined set as inputs and their binding affinities as labels. Then we measured each feature's importance using the data points' mean decrease in impurity (MDI) at that feature, where the impurity is measured by Gini index [61].

We identified the features that were present at least once among the top 10 most important features across 100 random splits of the data. We found 77 features that satisfy this criterion. We also find that performance of a GBR trained on these 77 features is comparable

to the performance of a GBR trained on the original 1.2 million features of the flattened initial images (Table B in appendix). Because most of these features will be pruned regardless during the next step (iterative ablation), we decided that it is sufficient to proceed into the next step using only these features.

B.2 Iterative ablation.

Starting with these 77 features, we performed an iterative procedure to identify and ablate the least important features: At each iteration, we ablated the feature that when deleted, GBRs trained on the remaining features had the lowest root mean squared error (RMSE) across 50 random data splits. We term this procedure the *ablate-and-test* procedure. Code for ablate-and-test follows this paragraph, and the accuracies of optimal GBRs trained on each number of features from 77 to 1 are shown in Section B.3. The resulting set of 10 features is termed the *persistence fingerprint*.

B.2.1 Algorithm for Iteratively Ablating Features.

Following is the algorithm that takes in the 77-dimensional vector of features (`highly_selected_observations`) resulted from Section B.1, and iteratively deletes the feature that when deleted, results in models with the lowest RMSE across 10 runs.

```
from multiprocessing import Pool, cpu_count
import numpy as np

assert highly_selected_observations.shape[1] == len(highly_selected_observations_indices)  # Should both be 77

ablation_iter_results = []  # Keeps track of results for each iteration of ablation

# ablate one element at a time
for iter in range(highly_selected_observations.shape[1] - 1):
    def ablate_run(index):
        ablated_observations = np.delete(highly_selected_observations, index, axis=1)
        ablation_result = train_and_test(ablated_observations, binding_affinities, num_runs=10)
        return ablation_result

    with Pool(cpu_count() - 4) as p:
        ablation_results = p.map(ablate_run, range(highly_selected_observations.shape[1]))
```

```python

    # Find index with lowest RMSE (and thus the least important feature)
    min_rmse_index = np.argmin([r['rmse']['mean'] for r in ablation_results])
    min_rmse = ablation_results[min_rmse_index]['rmse']['mean']
    min_rmse_std = ablation_results[min_rmse_index]['rmse']['std']

    print(f'Finished iteration {iter}, deleted index {min_rmse_index} (original index {highly_selected_observations_indices[min_rmse_index]}), new RMSE: {min_rmse:.4f} +/- {min_rmse_std:.4f}')

    # Delete that index
    highly_selected_observations = np.delete(highly_selected_observations, min_rmse_index, axis=1)
    highly_selected_observations_indices = np.delete(highly_selected_observations_indices, min_rmse_index)

    ablation_iter_results.append({
        'min_rmse_index': min_rmse_index,
        'iteration_results': ablation_results[min_rmse_index],
        'remaining_indices': highly_selected_observations_indices
    })
```

B.3 Final and Intermediate Results from Iterative Ablation

See Table B for performances of gradient boosting regressors with 1.2M, 77 and 10 features respectively, all with 100 trees. See Figures D and E for the accuracy of optimal GBRs with respect to the number of input features and number of trees, respectively. After balancing interpretability with performance, we selected the vector with 10 features to be the *persistence fingerprint* and a regressor with 13 regression trees.

B.3.1 What if we used a different number of features?

Refining gradient boosted tree ensembles into PATH$^+$ required two important parameters: Number of trees in the ensemble and number of features. In this section, we evaluate the accuracy of PATH$^+$ if a different number of features is used.

We first confirmed that 13 is still an optimal number of trees for different number of features by train-test split on the PDBBind dataset (Fig F). Then we evaluated the performance of gradient boosted ensembles trained on the top 5, 10, 20, 40, 70 features on orthogonal datasets. We find the increasing the number of features to 20 or 40 marginally improves the RMSE on orthogonal datasets.

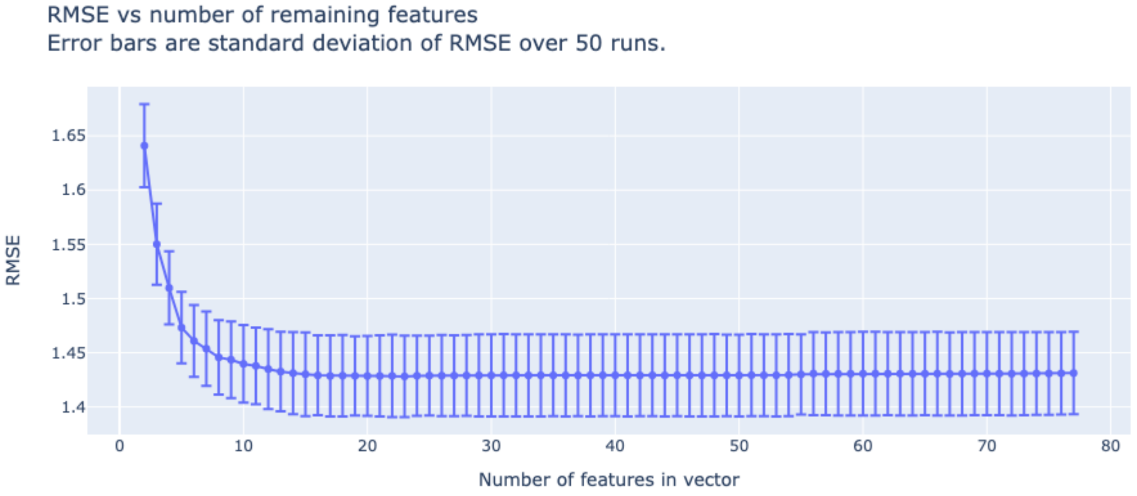

Figure D: **RMSE of trained GBRs with respect to number of features (from 77 to 1) in the vector through the iterative refinement process.** We have chosen the number of features to be 10, as we observed that 10 is the number at which the performance of trained GBRs start to degrade quickly. As a result, this vector with 10 features (components) is termed *persistence fingerprint*. In this plot, the tree depth is set to be 3, learning rate is set to be 0.3, and number of estimators (trees) is set to be 100.

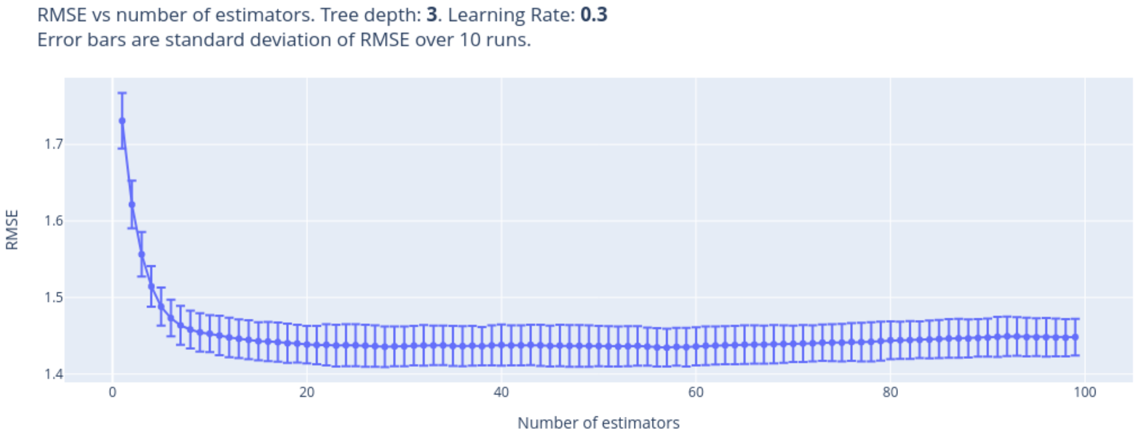

Figure E: **RMSE of trained GBRs with respect to the number of trees, using persistence fingerprint as input, with tree depth 3 and learning rate 0.3**. These tree depth and learning rate were determined using a hyperparameter search for interpretability and performance. We selected 13 as the number of trees after balancing interpretability and performance.

Table B: **Performance of intermediate GBR models across 100 random restarts (except TNet-BP as reported in [43]) on PDBBind v2020 refined set shows that intermediate ablated models did not lose much performance.** The values are reported in $pK_i = -\log_{10} K_i$ or $pK_d = -\log_{10} K_d$, as is done in PDBBind [50]. All values measured by us are reported in mean ± standard deviation across 100 random restarts. Train:Test ratio is 9-to-1.

**Model architecture**	TNet-BP (as reported in [43])	TNet-BP (our implementation)	GBR	GBR	GBR
**Number of features**	14472	14472	1.2M (flattened initial images)	77 (after ablation by MDI)	10 (after ablate-and-test i.e.,persistence fingerprint)
**RMSE**	1.37	1.69 ± 0.08	1.36 ± 0.04	1.44 ± 0.04	1.45 ± 0.04
**Pearson**	0.83	0.56 ± 0.04	0.72 ± 0.02	0.67 ± 0.02	0.67 ± 0.02

Due to the coincidence that all of the top 77 features selected through MDI can be interpreted under the language of IPCs, if one opts to train using more than 10 features, the final ensemble model will still enjoy the interpretability and fast compute algorithm. See section F for details.

B.4 Results for Alternative Regression Methods on the 10 Selected Features

To confirm that decision trees are the optimal model choice on the final curated features, we used LazyPredict [118] and trained the selected 10 features (i.e., persistence fingerprint) on 41 other regressors. We found that gradient boosting regressor still performs reasonably well among other regressors. The results are shown in Table C.

B.5 Representability of persistence fingerprint

To supplement Fig 1 in the Main MS, which shows the representability of persistence fingerprints with PaCMAP [52], Fig H shows boxplots where each boxplot represents a different component of the persistence fingerprint, and each data point represents a protein-ligand complex in the PDBBind v2020 refined set. This figure shows the distribution of magni-

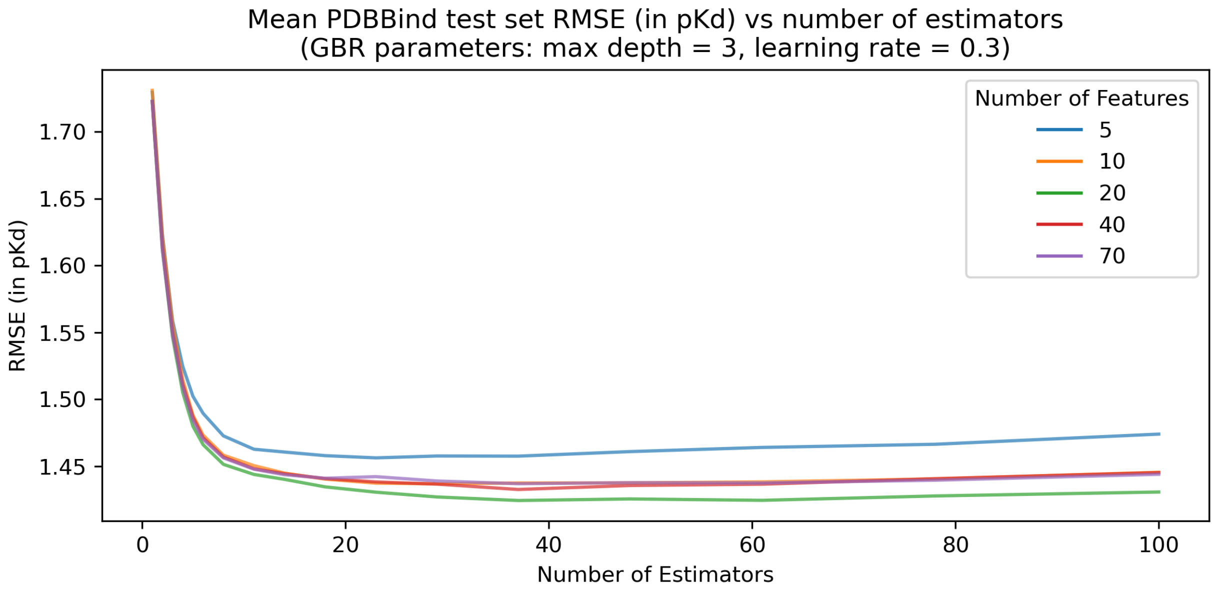

Figure F: **13 is still an optimal number of trees for different number of features.** The GBR parameters (max depth = 3, learning rate = 0.3) are from a hyperparameter search before (See caption of Fig D).

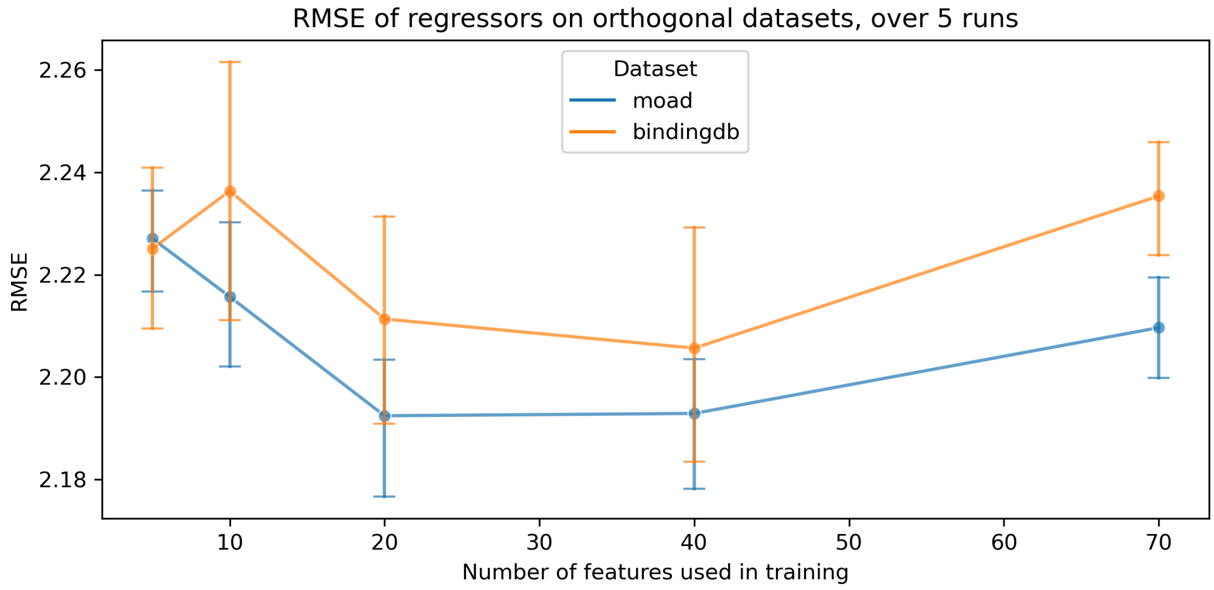

Figure G: **Increasing the number of features from 10 to 20 or 40 marginally improves performance of trained regressor on orthogonal datasets.** The number of regression trees is fixed at 13. Error bars: 95% confidence interval.

Table C: **Results for training and testing the selected 10 features (i.e. persistence fingerprint) on other regression methods across 100 random restarts shows that regression trees are still the most accurate (with lowest RMSE) models on the persistence fingerprint representation.** Each entry of the table shows mean ± standard deviation. All results are reported in $pK_i = -\log_{10} K_i$ or $pK_d = -\log_{10} K_d$. As in Table B, the dataset is PDBBind v2020 refined set. Train:Test ratio is 9-to-1. All models are run in their default parameters in the LazyPredict [118] package.

	RMSE across 100 restarts	R^2 across 100 restarts	Adjusted R^2 across 100 restarts
ExtraTreesRegressor	1.38 ± 0.04	0.49 ± 0.03	0.48 ± 0.03
RandomForestRegressor	1.40 ± 0.04	0.47 ± 0.04	0.46 ± 0.04
LGBMRegressor	1.42 ± 0.04	0.46 ± 0.03	0.45 ± 0.04
HistGradientBoostingRegressor	1.43 ± 0.05	0.46 ± 0.03	0.45 ± 0.04
NuSVR	1.43 ± 0.05	0.45 ± 0.03	0.44 ± 0.04
SVR	1.43 ± 0.05	0.45 ± 0.04	0.44 ± 0.04
MLPRegressor	1.44 ± 0.05	0.45 ± 0.04	0.43 ± 0.04
GradientBoostingRegressor	1.44 ± 0.04	0.44 ± 0.03	0.43 ± 0.03
KNeighborsRegressor	1.47 ± 0.05	0.42 ± 0.04	0.41 ± 0.04
BaggingRegressor	1.47 ± 0.05	0.42 ± 0.04	0.41 ± 0.04
XGBRegressor	1.48 ± 0.05	0.41 ± 0.04	0.40 ± 0.04
AdaBoostRegressor	1.51 ± 0.04	0.39 ± 0.03	0.38 ± 0.03
BayesianRidge	1.56 ± 0.05	0.35 ± 0.03	0.34 ± 0.04
ElasticNetCV	1.56 ± 0.05	0.35 ± 0.03	0.34 ± 0.03
RidgeCV	1.56 ± 0.05	0.35 ± 0.03	0.34 ± 0.04
LassoCV	1.56 ± 0.05	0.35 ± 0.03	0.34 ± 0.04
Ridge	1.56 ± 0.05	0.35 ± 0.03	0.34 ± 0.04
TransformedTargetRegressor	1.56 ± 0.05	0.35 ± 0.03	0.34 ± 0.04
LinearRegression	1.56 ± 0.05	0.35 ± 0.03	0.34 ± 0.04
LassoLarsCV	1.56 ± 0.05	0.35 ± 0.03	0.34 ± 0.04
LassoLarsIC	1.56 ± 0.05	0.35 ± 0.03	0.34 ± 0.04
HuberRegressor	1.56 ± 0.05	0.35 ± 0.04	0.34 ± 0.04
SGDRegressor	1.56 ± 0.05	0.35 ± 0.03	0.34 ± 0.04
LinearSVR	1.56 ± 0.05	0.35 ± 0.04	0.34 ± 0.04
OrthogonalMatchingPursuitCV	1.56 ± 0.05	0.35 ± 0.03	0.33 ± 0.04
TweedieRegressor	1.58 ± 0.05	0.34 ± 0.03	0.32 ± 0.03
PoissonRegressor	1.58 ± 0.05	0.33 ± 0.04	0.32 ± 0.04
GammaRegressor	1.59 ± 0.05	0.32 ± 0.03	0.31 ± 0.03
LarsCV	1.65 ± 0.23	0.26 ± 0.29	0.25 ± 0.29
OrthogonalMatchingPursuit	1.65 ± 0.05	0.27 ± 0.04	0.25 ± 0.04
ElasticNet	1.73 ± 0.05	0.20 ± 0.02	0.18 ± 0.02
Lasso	1.93 ± 0.05	0.00 ± 0.00	-0.01 ± 0.01
LassoLars	1.93 ± 0.05	0.00 ± 0.00	-0.01 ± 0.01
DummyRegressor	1.94 ± 0.05	-0.00 ± 0.00	-0.02 ± 0.00
DecisionTreeRegressor	1.99 ± 0.06	-0.06 ± 0.08	-0.08 ± 0.08
ExtraTreeRegressor	2.01 ± 0.06	-0.08 ± 0.09	-0.10 ± 0.09
RANSACRegressor	2.08 ± 0.21	-0.17 ± 0.25	-0.19 ± 0.25
Lars	2.55 ± 0.76	-0.90 ± 1.11	-0.93 ± 1.13
PassiveAggressiveRegressor	2.59 ± 0.60	-0.87 ± 0.92	-0.91 ± 0.94
GaussianProcessRegressor	3.63 ± 0.39	-2.57 ± 0.84	-2.64 ± 0.85
KernelRidge	6.60 ± 0.07	-10.64 ± 0.64	-10.87 ± 0.65

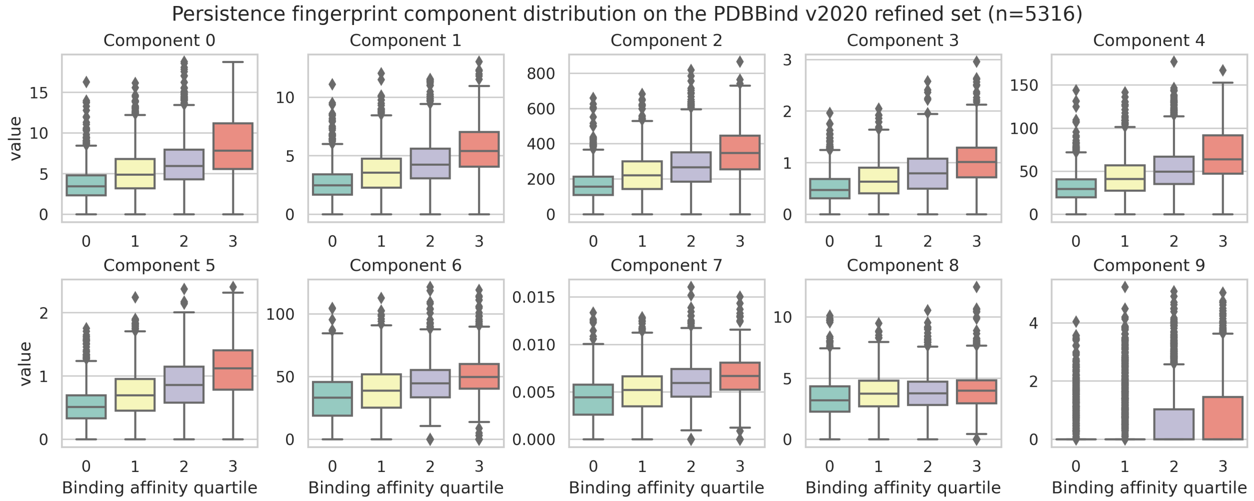

Figure H: **Persistence fingerprint has discriminating power on binding affinity.** Boxplots of each persistence fingerprint component with respect to different quartiles of binding affinity on the PDBBind v2020 refined set show that binding affinity correlates with the values of persistence fingerprint components. The x-axis and color correspond to quartiles of data by their experimental binding affinity. The y-axis corresponds to the values of at that certain persistence fingerprint component.

tude of each component of persistence fingerprint for protein-ligand complexes, grouped in quartiles by their experimentally-measured binding affinities. Fig I show boxplots for the Binding MOAD and BindingDB datasets produced using the same methodology.

As expected, persistence fingerprint shows discriminating power on the PDBBind v2020 refined set (the training set). The remarkable observation is that the discriminating power of persistence fingerprint remains in the Binding MOAD and BindingDB datasets, which consist of more protein-ligand complexes than PDBBind v2020 refined set and the complexes are selected using different methodologies from PDBBind, albeit the discriminating power is to a lesser extent for components 8 and 9 of the fingerprint (Figures H and I).

We also measured the generalizability of persistence fingerprint beyond the training dataset (Fig 1). We computed persistence fingerprints on two protein-ligand binding affinity datasets from the BioLiP database [131, 53], namely Binding MOAD [44, 45, 46, 47] and BindingDB [48, 49], which consist of protein-ligand complexes with binding affinity (ΔG) in the range -2.7 to -16.3 kcal/mol.

To visualize the distribution of persistence fingerprint, we used PaCMAP [52], a recent dimensionality reduction technique that down-projects higher dimensional data points onto a two-dimensional manifold, similar to UMAP [132] and t-SNE [133], which additionally preserves "both local and global structure of the data in original space". We used PaCMAP [52] to down-project persistence fingerprints of the protein-ligand complexes in the PDBBind

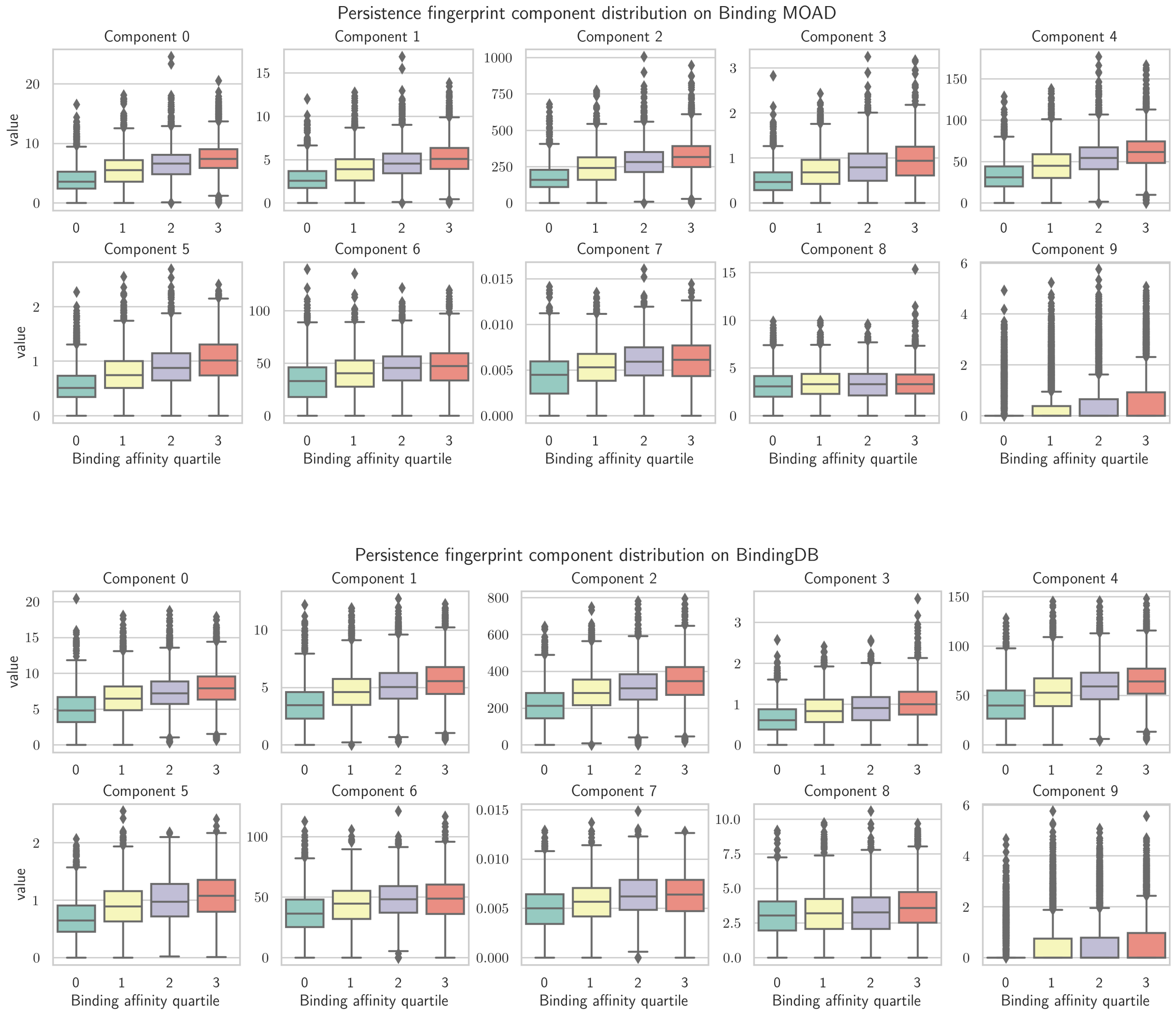

Figure I: **Boxplots of the persistence fingerprint values with respect to different quartiles of binding affinity on the Binding MOAD and BindingDB datasets show that the discriminating power of persistence fingerprint generalizes beyond its training dataset (which is PDBBind v2020 refined set, as seen in Fig H), albeit to a lesser extent for components 8 and 9 of the persistence fingerprint.** The x-axis and color correspond to quartiles of data by their experimental binding affinity. The y-axis corresponds to the values of at that certain persistence fingerprint component.

v2020 refined set [50] (training set), Binding MOAD , and BindingDB datasets. The results are shown in Fig 1. In these plots, each point represents a protein-ligand complex in the dataset, the position of each point is the projection of its persistence fingerprint, and points are colored according to its experimental binding affinity. In Fig 1, we see that binding affinity is separated by persistence fingerprint, as evidenced by a gradient of colors, meaning that complexes with similar binding affinities are similar to each other in persistence fingerprint. These plots show that the representational power of persistence fingerprints generalizes beyond its dataset.

B.6 Performance of PATH$^+$ in Different Ligand Types in BioLiP

To test the generalizability of PATH$^+$, we report its performance across different ligand types in BioLiP (Binding MOAD + BindingDB) in Fig J.

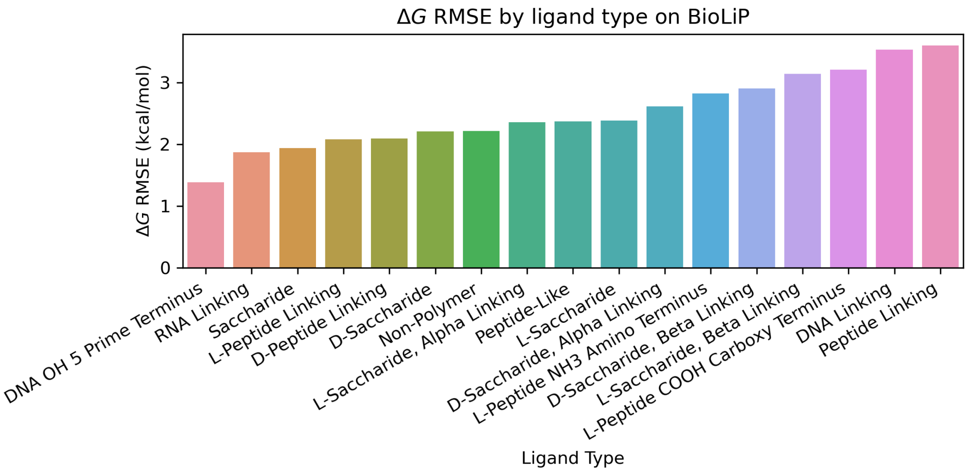

Figure J: **The performance of PATH$^+$ across different ligand types.** Shown: Root mean squared error of PATH$^+$'s predicted ΔG (kcal/mol) across different ligand types in BioLiP. The ligand types are as annotated by the PDB Chemical Component Dictionary [117].

C Computational complexity of persistence fingerprint

C.1 Computational complexity of IPCs

Persistent homology is computationally expensive to find. In particular, the k-skeleton of a Rips complex (a subcomplex with simplex dimension up to k) has $\mathcal{O}(n^{k+1})$ simplices, where n is the number of points in the input point cloud [100]. The time complexity to

compute persistent homology is then $\mathcal{O}\left(\left(n^{k+1}\right)^{\omega}\right) = \mathcal{O}\left(n^{(k+1)\omega}\right)$, where $\omega \approx 2.4$ is the matrix multiplication exponent [134, 114, 135]. For a protein-ligand complex, let m be the number of ligand atoms and n be the number of protein atoms. When computing the IPCs from whence persistence fingerprint is derived, both cases for $k \in \{0, 1\}$ need to be computed and the computational complexity is $\mathcal{O}((m+n)^{4.8})$. Previous binding affinity prediction algorithms [36, 43, 34] that use persistent homology all calculated the the full persistence diagram for at least $k \in \{0, 1\}$, hence they all have computational complexity at least $\mathcal{O}((m+n)^{4.8})$.

Below in Sections C.2 and C.3, we show an effective algorithm to compute a provable approximation of persistence fingerprint.

C.2 Computational complexity of our provably ε-accurate approximation algorithm for persistence fingerprint is independent of protein size

By Section C.1, constructing the full IPC for identifying the components of persistence fingerprint naively takes $\mathcal{O}(n^{4.8})$ time for each protein-ligand complex, where n is the number of atoms in the protein-ligand complex. However, in many biologically relevant protein-ligand complexes [57, 58, 59], n can be very large, resulting in unwieldy runtime and space consumption by these algorithms. In this subsection, we prove Theorem 1:

Theorem 1 (Complexity of approximating persistence fingerprint)**.** *Assume there exists a fixed lower bound on interatomic distances in a protein-ligand complex. Let the number of protein atoms be n, the number of ligand atoms be m, and $\omega \approx 2.4$ be the matrix multiplication exponent [114]. For any $0 < \varepsilon < 1$, after an $\mathcal{O}(mn\log(mn))$ preprocessing procedure, we can compute an approximation to the persistence fingerprint in $\mathcal{O}(m\log^{6\omega}(m/\varepsilon))$ time, independent of protein size, such that the maximum difference between each component in this approximation and that of the corresponding element in the true persistence fingerprint is less than ε.*

First, recall that in the hard sphere model[4], each atom is modeled by a hard sphere with the atom's van der Waals radius as the sphere's radius. Let r_m be the smallest van der Waals radius among all atoms. Let $\eta = \pi/\sqrt{18} \approx 0.74$ be the maximum packing density of congruent spheres in three dimensions [136]. Then if we denote a to be the number of atoms in a ball of radius r, we have $a\pi r_m^3 \leq 4\pi r^3/\eta$. Let $N(r) = \frac{1}{\eta}\left(\frac{r}{r_m}\right)^3$ be the maximum number

[4]We use the hard sphere model for simplicity of calculation. The result will be identical if we used, say, a space-filling model.

of atoms that can be packed within a ball of radius r such that each atom's "hard sphere" is in this ball of radius r, we have $a \leq N(r)$.

We claim that we can remove protein atoms that are more than a certain distance r_ε from the ligand while erring from the true value of that component by less than ε, and r_ε can be set independent of the protein size or protein shape. This removal will reduce the computation time of persistence fingerprint to become independent of protein size. In the following proof, we only prove the case for the persistence fingerprint components that result from 1D IPCs, and the proof for removing atoms in optimizing 0D IPC is easily obtained *mutatis mutandis.*

Suppose we remove (i.e., prune) all protein atoms beyond a certain radius R of a certain ligand atom $l \in L$, we want to find the resulting difference in the contribution to a component of the persistence fingerprint, which is $\int\limits_I \gamma(x)dx$ over a certain closed interval $I = [i_l, i_r] \subset \mathbb{R}$. This interval I is given by the table of features of persistence fingerprint (Table 1) and has a fixed width $\Delta i = i_r - i_l = 0.5$. Fix l. Define the protein atoms within the radius r be $P_r = \{p \in P : d(p,l) \leq r\}$ and denote the protein atoms to be pruned $P'_r = P - P_r$. Additionally, provide the constraint that $r > i_r$. Then for a given atom $p' \in P'_r$, the contribution of p' in this integral, denoted $c(p')$ with $c : P \to \mathbb{R}$, satisfies

$$c(p') \leq \overline{\overline{J}} \int\limits_I g_{d(p',l)}(x)\mathrm{d}x \tag{7}$$

where

$$J = \{(p',l) : (p',l) = \underset{e \in E}{\operatorname{argmax}}\, w(e), (V,E) \in H_1(\mathbf{VR}(S)_{\max R})\} \tag{8}$$

is the set of 1-cycles whose longest edge is the (p',l), and where each generator of the first homology group, a 1-cycle, is viewed as a bipartite graph (Fig C). Eq. (7) follows from Lemma 2. For convenience, let $||p'|| = d(p',l)$ and $G(r) = \int\limits_I g_r(x)dx$.

Lemma 3 (Maximum number of 1D holes from opposition distance)**.** *Consider the Vietoris-Rips filtration of a protein-ligand complex with the opposition distance* d_{op}*. Let the set of protein atoms be* P *and the set of ligand atoms be* L*. Suppose* $\overline{\overline{P}} \geq 2$ *and* $\overline{\overline{L}} \geq 2$*. Then at any filtration parameter* $r \geq \sup\limits_{p \in P, l \in L} d(p,l)$*, the homology group in the simplicial complex of the filtration with parameter* r *has rank* $(\overline{\overline{P}} - 1)(\overline{\overline{L}} - 1)$*.*

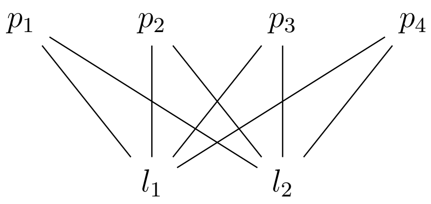

Figure K: Example of K for $P = \{p_1, p_2, p_3, p_4\}$ and $L = \{l_1, l_2\}$.

Proof of Lemma 3: Let the simplicial complex at $r \geq \sup_{p \in P, l \in L} d(p, l)$ be $K = \mathbf{VR}(S)_r$. Then K as a vector space has basis $\{[p_i, l_j] : p_i \in P, l_j \in L\} \cup \{[p_i] : p_i \in P\} \cup \{[l_j] : l_j \in L\}$. Consider, for example, the case where $\overline{\overline{P}} = 4$ and $\overline{\overline{L}} = 2$. Then K looks like

By Lemma 1, $H_1(K) \cong Z_1(K) = \ker(\partial_1(K))$. And

$$\ker(\partial_1(K)) = \ker(\partial(\lambda_{11}[p_1, l_1] + \lambda_{12}[p_1, l_2] + ... + \lambda_{42}[p_4, l_2])) \tag{9}$$

$$= \ker(\lambda_{11}[l_1] - \lambda_{11}[p_1] + \lambda_{12}[l_2] - \lambda_{12}[p_2] + ... + \lambda_{42}[l_2] - \lambda_{42}[p_4]) \tag{10}$$

where λ_{ij} is the coefficient of the generator $[p_i, l_j]$. We are led to solve the following system of equations:

$$\begin{cases} -\lambda_{11} + -\lambda_{12} = 0 \\ -\lambda_{21} + -\lambda_{22} = 0 \\ -\lambda_{31} + -\lambda_{32} = 0 \\ -\lambda_{41} + -\lambda_{42} = 0 \\ \lambda_{11} + \lambda_{21} + \lambda_{31} + \lambda_{41} = 0 \\ \lambda_{12} + \lambda_{22} + \lambda_{32} + \lambda_{42} = 0. \end{cases} \tag{11}$$

In this system of equations (Eq. (11)), the first four equations are generated by each of the protein atoms and the last two equations are generated by each of the ligand atoms. Note that exactly one equation is redundant, so this system of equations yields 5 linearly independent constraints. Since there are 8 variables, given 5 constraints then there are $8 - 5 = 3$ free variables, which is the rank of $H_1(K)$.

This reasoning generalizes to any arbitrary number of protein atoms and ligand atoms. Suppose K has n protein atoms and m ligand atoms, then

$$\ker(\partial_1(K)) \tag{12}$$

$$= \ker\left(\partial_1\left(\sum_{i=1}^{n}\sum_{j=1}^{m}\lambda_{ij}[p_i, l_j]\right)\right) \tag{13}$$

which generates the system of equations

$$\begin{cases} \{\sum_{j=1}^{m} -\lambda_{ij} = 0\}_{i=1}^{n} \\ \{\sum_{i=1}^{n} \lambda_{ij} = 0\}_{j=1}^{m} \end{cases} \tag{14}$$

Similar to before, this system of $m+n$ equations has exactly one redundant equation. Subtracting the number of constraints from the number of variables, we get that the number of free variables to be $mn - (m+n-1) = (m-1)(n-1)$, which is the rank of $H_1(K)$. □

Hence

$$\overline{\overline{J}} \leq \mathrm{rank}(H_1(\mathbf{VR}(P_{||p'||} \sqcup L)_{\max R})) \tag{15}$$

$$\leq \left(\overline{\overline{P_{||p'||}}} - 1\right)\left(\overline{\overline{L}} - 1\right) \tag{16}$$

$$\leq N\left(||p'||\right)\overline{\overline{L}} \tag{17}$$

where the protein-ligand complex after pruning all protein atoms farther from l than p' is denoted $P_{||p'||} \sqcup L$. The inequality of Eq. (15) follows from the fact that a 1-cycle formed with any protein atom $p_\times \in P'_{||p'||}$ must have its longest edge longer than $d(p', l)$ because $d(p_\times, l) > d(p', l)$. The inequality of Eq. (16) follows from Lemma 3.

We can simplify Eq. (7):

$$c(p') \leq \overline{\overline{L}} N(||p'||) G(||p'||). \tag{18}$$

Now we have a bound on $c(p')$. Then, the total effect of all the atoms pruned out on the persistence fingerprint component of concern is given by

$$\sum_{p' \in P'_r} c(p') \leq \sum_{p' \in P'_r} \overline{\overline{L}} N(||p'||) G(||p'||) \tag{19}$$

where $G(r) = \int_I g_r(x)\mathrm{d}x$ is the integral of g_r is the Gaussian function centered at r with standard deviation σ, integrated over the interval $I = [i_l, i_r]$.

Lemma 4 (Monotonicity and bound of $G(r)N^2(r)$)**.** *There exists r_0 such that for all $r > r_0$,*

1. *$G(r)N^2(r)$, $G(r)N(r)$, and $G(r)$ monotonically decrease.*
2. *$G(r)N^2(r) < \exp(-r)$.*

and r_0 does not depend on ε, $\overline{\overline{P}}$, or $\overline{\overline{L}}$.

Proof of Lemma 4: Recall that $N(r)$ is a cubic polynomial in r. Intuitively, $g_r(x)$ decays exponentially and is integrated over an interval $[i_l, i_r]$ with a fixed width Δi to yield G, so asymptotically as $r \to \infty$, $G(r)N^2(r)$ behaves like $\frac{r^6}{e^{2r}}$, which tends to 0 and decays faster than $\exp(-r)$. To be precise, consider any $r > i_r$, we have

$$\begin{aligned} G(r) &= \int_I g_r(x)\mathrm{d}x && (20) \\ &\leq (i_r - i_l) g_r(i_l) && (21) \\ &= \frac{\Delta i}{\sigma\sqrt{2\pi}} \exp\left(-\frac{1}{2}\left(\frac{i_r - r}{\sigma}\right)^2\right) && (22) \end{aligned}$$

where the inequality in Eq. (21) follows from the assumption that $r > i_r$ and hence $g_r(x)$ is monotonically decreasing in I.

Then to show that there exists r_0 such that $G(r)N^2(r) < 0$ is strictly decreasing beyond r_0:

$$
\begin{aligned}
&\frac{\mathrm{d}}{\mathrm{d}r} G(r) N^2(r) && (23)\\
=&\frac{\mathrm{d}}{\mathrm{d}r}\left(\frac{r^6}{\eta^2 r_m^3}\frac{\Delta i}{\sigma\sqrt{2\pi}}\exp\left(-\frac{1}{2}\left(\frac{i_r - r}{\sigma}\right)^2\right)\right) && (24)\\
=&\frac{\mathrm{d}}{\mathrm{d}r}\left(\alpha r^6 \exp\left(-\frac{1}{2}\left(\frac{i_r - r}{\sigma}\right)^2\right)\right) && (25)\\
=&-\alpha\sigma^{-2}\left(r^5(r^2 - r i_r - 6\sigma^2)\exp\left(-\frac{(i_r - r)^2}{2\sigma^2}\right)\right) && (26)
\end{aligned}
$$

where $\alpha = \frac{\Delta i}{\eta^2 r_m^3 \sigma\sqrt{2\pi}}$ is a positive constant. Since $\alpha\sigma^{-2}$ and $\exp\left(-\frac{(i_r - r)^2}{2\sigma^2}\right)$ are positive and there exists r_1 such that $r^5(r^2 - r i_r - 6\sigma^2)$ is positive for all $r > r_1$, we have that Eq. (26) is negative for all $r > r_1$. In other words, $G(r)N^2(r)$ monotonically decreases beyond r_1.

Similarly, there exists r_2 such that $G(r)N(r)$ monotonically decreases for all $r > r_2$. Thus choosing $r > \max(r_1, r_2)$ satisfies the first claim of Lemma 4.

To show the second claim in Lemma 4, we first show that there exists r_3 such that for all $r > r_3$, we have

$$
\begin{aligned}
\alpha r^6 \exp\left(-\frac{1}{2}\left(\frac{i_r - r}{\sigma}\right)^2\right) &< \exp(-r) && (27)\\
\log\alpha + 6\log r + \left(-\frac{1}{2}\left(\frac{i_r - r}{\sigma}\right)^2\right) &< -r && (28)\\
-\frac{1}{2\sigma^2} r^2 + \left(1 + \frac{i_r}{\sigma} r\right) + 6\log r + \log\alpha &< 0. && (29)
\end{aligned}
$$

Since the left hand side of Eq. (29) is dominated by $-\frac{1}{2\sigma^2} r^2$, there exists r_3 such that this inequality is true for any $r > r_3$, which satisfies the second claim of Lemma 4.

Finally note that i_r, r_1, r_2, r_3 depend only on the integration interval I in $G(r)$, and not on ε, $\overline{\overline{P}}$, or $\overline{\overline{L}}$. Therefore, for $r_0 = \max(i_r, r_1, r_2, r_3)$, Lemma 4 is satisfied. □

Choose a pruning radius $r > r_0$. We claim that for any number and any placement of protein atoms, the total effect of pruning out all atoms in P'_r, as described in 19, is finite.

Consider a partition of $[r, \infty)$: $\{r_i\}_{i=0}^{\infty} \subset \mathbb{R}$ starting from a pruning radius r such that $r = r_0 \le r_1 \le r_2 \le \ldots$ and $\forall i \in \mathbb{Z}^+ : r_{i+1} - r_i = \Delta r$ for some fixed value Δr. Then note that in each interval $[r_i, r_{i+1}]$, there can at most be $N(r_{i+1}) - N(r_i)$ protein atoms. Then the total contribution of atoms in $[r_i, r_{i+1}]$ to our persistence fingerprint can be bounded component:

$$\sum_{\{p':||p'||\in[r_i,r_{i+1}]\}} c(p') \tag{30}$$

$$\leq \sum_{\{p':||p'||\in[r_i,r_{i+1}]\}} \overline{\overline{L}} N(||p'||)G(||p'||) \tag{31}$$

$$\leq \sum_{\{p':||p'||\in[r_i,r_{i+1}]\}} \overline{\overline{L}} N(r_i)G(r_i) \tag{32}$$

$$\leq \overline{\overline{L}}(N(r_{i+1}) - N(r_i))N(r_i)G(r_i). \tag{33}$$

Then the total contribution of all pruned atoms (i.e., P'_r) to our persistence fingerprint component is

$$\sum_{p'\in P'_r} c(p') = \sum_{i=0}^{\infty} \sum_{\{p':||p'||\in[r_i,r_{i+1}]\}} c(p') \tag{34}$$

$$\leq \sum_{i=0}^{\infty} \overline{\overline{L}}(N(r_{i+1}) - N(r_i))G(r_i)N(r_i) \tag{35}$$

$$\leq \sum_{i=0}^{\infty} \overline{\overline{L}} N(r_{i+1})G(r_i)N(r_i). \tag{36}$$

By assumption, $1/G(r) \in \mathcal{O}(e^r)$ and recalling that $N(r) \in \mathcal{O}(r^3)$, the last sum (i.e., Eq. (36)) converges. In other words, for any pruning radius r, the error resulting from pruning all protein atoms beyond r is bounded. Since the pruning error monotonically decreases with respect to increasing r, we conclude that given any ε, there exists r_ε such that for any pruning radius $r > r_\varepsilon$, the error in calculating a persistence fingerprint component of removing all atoms in P'_r will be less than ε, independent of the protein size.

We just showed that for a given ligand atom l and a given persistence fingerprint component, there exists a pruning radius r_ε independent of protein size such that when all protein atoms farther than r_ε from l are removed, the error in the contribution of l in this persistence fingerprint component is less than ε. Since each persistence fingerprint component is a sum of contributions of each ligand atom, we can extend our result easily. Let $P'_\varepsilon(l) = P - P_\varepsilon(l)$ be the set of protein atoms that can be pruned for a given ligand atom $l \in L$ with an error less than ε. Then for a ligand L, $\bigcap_{l\in L} P'_{\varepsilon/\overline{\overline{L}}}(l)$ can be pruned while guaranteeing a total pruning error of less than ε. The remaining atoms after pruning are contained in a finite volume, thus there is an upper bound for the number of atoms. This bound on pruning radius is only dependent on the number of ligand atoms and ε, which guarantees an upper bound on the

runtime of persistence fingerprint for a fixed ligand and an ε.

C.3 Computational complexity with respect to ligand size and ε

In Section C.2, we showed that using the approximation scheme we devised, complexity of computing the persistence fingerprint can be independent of protein size. In this subsection, we derive computational complexity of this approximation of persistence fingerprint in terms of the number of atoms in the ligand $\overline{\overline{L}}$ and the error bound ε. First, following Eq. (36), we have that for a certain ligand atom and a certain set of protein atoms P'_r to be removed, the total pruning error is bounded by

$$\sum_{p' \in P'_r} c(p') \leq \sum_{i=0}^{\infty} \overline{\overline{L}}(N(r_{i+1}) - N(r_i))G(r_i)N(r_i) \tag{37}$$

$$\approx \overline{\overline{L}} \int_r^{\infty} N(r)G(r)N(r)\mathrm{d}r, \tag{38}$$

where the approximation in Eq. (38) follows when $[R, \infty)$ is partitioned finely (i.e., $(r_{i+1} - r_i) \to 0$). Then given any ε bound on error, we want r_ε to satisfy

$$\overline{\overline{L}} \int_{r_\varepsilon}^{\infty} N(r)G(r)N(r)\mathrm{d}r < \varepsilon. \tag{39}$$

By Lemma 4, there is r_0 independent of L or ε such that for all $r > r_0$, we have $N(r)G(r)N(r) < \exp(-r)$. Then for any $r_\varepsilon > \max(r_0, \log(\overline{\overline{L}}/\varepsilon))$, we have

$$\overline{\overline{L}} \int_{r_\varepsilon}^{\infty} N(r)G(r)N(r)\mathrm{d}r \tag{40}$$

$$< \overline{\overline{L}} \int_{r_\varepsilon}^{\infty} \exp(-r)\mathrm{d}r \tag{41}$$

$$< \overline{\overline{L}} \exp(-r_\varepsilon) \tag{42}$$

$$< \varepsilon. \tag{43}$$

Therefore, any pruning radius $r_\varepsilon > \max(r_0, \log(\overline{\overline{L}}/\varepsilon))$ yields a pruning error of less than ε.

Then the number of atoms that are included to calculate the persistence fingerprint contribution of this particular ligand atom after pruning is at most $N(r_\varepsilon) = \mathcal{O}(\log^3(\overline{\overline{L}}/\varepsilon))$.

We must compute each ligand atom's contribution to the persistence fingerprint, and there are $\overline{\overline{L}}$ ligand atoms, so the runtime for computing the persistence fingerprint on this pruned complex is:

$$\mathcal{O}\left(\overline{\overline{L}}\left(\log^3\left(\overline{\overline{L}}/\varepsilon\right)\right)^{2\omega}\right) \tag{44}$$

$$=\mathcal{O}\left(\overline{\overline{L}}\log^{6\omega}\left(\overline{\overline{L}}/\varepsilon\right)\right) \tag{45}$$

where $\omega \approx 2.4$ is the matrix multiplication exponent [114], $\overline{\overline{L}}$ is the number of atoms in the ligand and ε is the error bound.

In summary, letting $n = \overline{\overline{P}}$, $m = \overline{\overline{L}}$, computing an ε-accurate approximation to each component of persistence fingerprint entails the following steps:

1. Preprocessing. First, compute and sort the pairwise distances between the protein and the ligand. Then, prune all protein atoms that are at least a distance r_ε away from any ligand atom. Preprocessing takes $\mathcal{O}(mn\log(mn))$ time due to sorting.

2. Compute the persistent homology of this pruned complex and calculate the persistence fingerprint. We have shown this approximation to be ε-accurate (Eq. (43)). This step takes $\mathcal{O}(m\log^{6\omega}(m/\varepsilon))$ time.

Since the number of components in the persistence fingerprint is constant (all 10 components are shown in Table 1 in the Main MS), the runtime of computing the entire persistence fingerprint is $\mathcal{O}(mn\log(mn))$ for preprocessing and pruning, and $\mathcal{O}(m\log^{6\omega}(m/\varepsilon))$ to compute the persistent homology of the pruned complex. ■

C.4 Empirical running times

Computation of persistence fingerprint using the pruning method described in previous sections was tested by calculating persistence fingerprint of protein-ligand complexes in the BioLiP dataset [53], which consists of entries from PDBBind [50], Binding MOAD [47], and Binding DB [48]. For this test, the pruning radius r is arbitrarily set to be 15 Angstroms.

Each trial in the runtime measurements was allocated a single core of an Intel Xeon processor and 16GB of RAM. The runtime showed a positive correlation with respect to the size of the ligand and is independent of size of the protein (Fig L. Pearson correlation coefficient (PCC) of protein size vs computation is -0.19. PCC of ligand size vs computation time is 1.00.), which corroborates Eq. (45). Overall, for the 48469 data points, the mean

was 41.35 seconds, standard deviation was 71.20 seconds, with a minimum of 1 second, 25% percentile at 19 seconds, 50% percentile at 26 seconds, 75% percentile at 34 seconds, and a maximum of 785 seconds.

Results for the accuracy of pruning is shown in Fig M, when compared to computing the full IPC and deriving persistence fingerprint from there. Note that the data points on this plot is a subset of the protein-ligand complexes in the BioLiP dataset can be computed by this approximation algorithm, since the full IPCs were intractable to compute for large protein-ligand complexes (Section C.1). Number of data points = 45199, mean = 3.16×10^{-11}, standard deviation = 2.87×10^{-9}, the values for the 25%, 50%, and 75% percentiles are all 0.0, with a max of 4.8×10^{-7}.

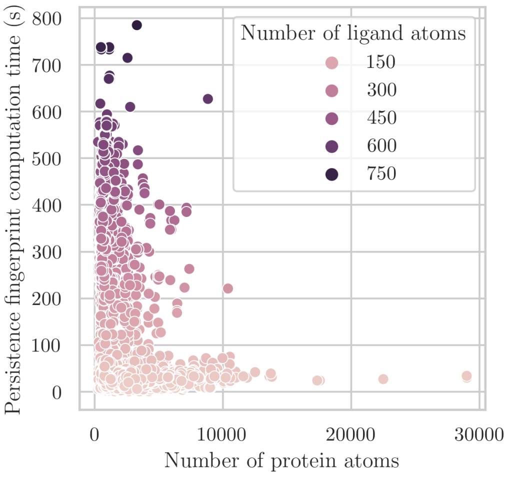

Figure L: **Runtime of computing persistence fingerprint on the BioLiP dataset corroborates Eq. (45) ($n = 48469$).** Pearson correlation coefficient (PCC) of protein size vs computation is −0.19, PCC of ligand size vs computation time is 1.00.

D The decision trees of PATH$^+$

D.1 Interpreting the decision trees

Given the small number of decision trees in PATH$^+$, we are able to plot and interpret each of the decision trees. The first decision tree is shown in Fig N.

Note that the nodes on the decision trees index into persistence fingerprint components with zero-based numbering. Also note the prediction values of the decision trees are expressed in pK_d or pK_i. For example, in this decision tree 1 (Fig N), the top node corresponds to the 5th component of the persistence fingerprint, which corresponds to the approximate number of 1-cycles formed by protein nitrogen atoms and ligand nitrogen atoms around 4

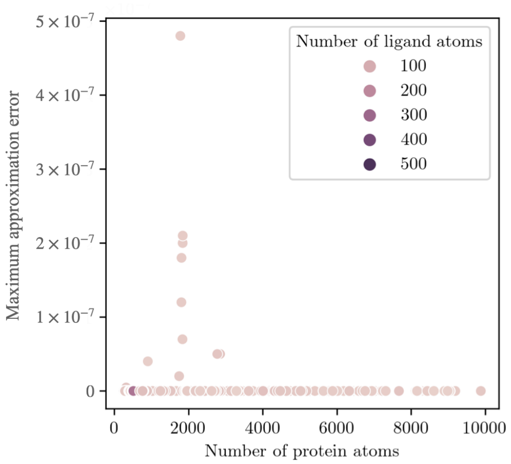

Figure M: **Error from pruning all atoms beyond radius r = 15 Å from all ligand atoms is extremely low.** Shown: scatter plot of pruning error with respect to number of protein atoms and number of ligand atoms. Note that the data points on this plot is only a subset of the entire BioLiP dataset, since the full IPCs were intractable to compute for large protein-ligand complexes (Section C.1). Number of data points = 45199, mean = 3.16×10^{-11}, standard deviation = 2.87×10^{-9}, the values for the 25%, 50%, and 75% percentiles are all 0.0, with a max of 4.8×10^{-7}.

Angstroms. A protein-ligand complex chooses one of the two branches based on a the value of that component.

As an example, two complexes are shown in Fig 9: HIV-1 protease in complex with VX-478 (PDB ID: 1hpv [115]) and humanised monomeric RadA in complex with indazole (PDB ID: 4b2i [116]). Looking at Fig N, we find that the 1hpv complex heads down the left branch because it has a higher value in the 5th component of its persistence fingerprint, which corresponds to a higher number of carbon-nitrogen bipartite matching around 10 Angstroms, while the 4b2i complex heads down the right branch due to a lower value in the 5th component of its persistence fingerprint.

A final prediction is done using all decision trees from persistence fingerprint.

Remarkably, through a subsequent literature review, we discovered the features in persistence fingerprint, which were completely automatically derived, are similar to the "interaction fingerprints" manually constructed in previous works on binding affinity prediction [97, 98]. Interpretability of PATH provides verification on the robustness beyond simply benchmarking on datasets and provides insights on the geometric features important to predicting binding affinity with persistent homology.

See the full set of decision trees in Section D.2.

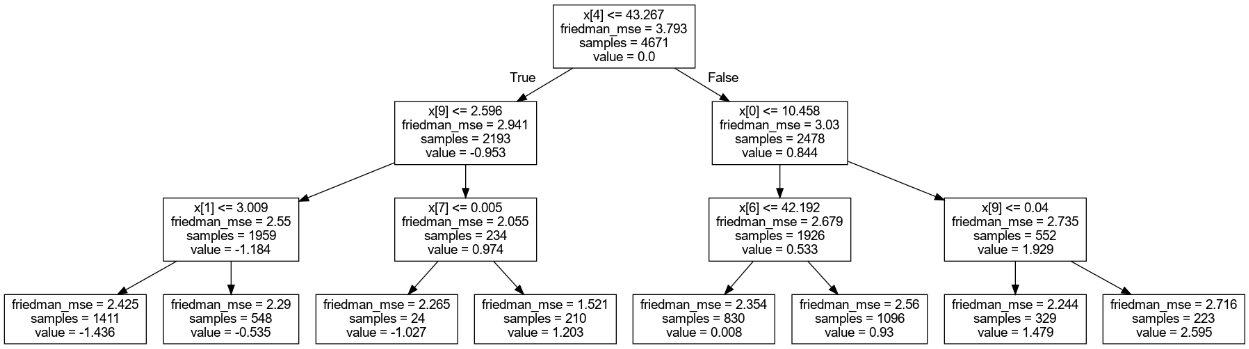

Figure N: **An example decision tree in PATH$^+$:** Decision tree 1 of 13 in PATH$^+$. The "x" at each node is the persistence fingerprint vector, and the indexing which follows is 0-based. For example, the topmost node reads "x[4]". We can refer to Table 1 to find this corresponds to the nitrogen-carbon bipartite matching at a distance of 10 Å. Given a protein-ligand complex that we wish to predict the binding affinity of, the decision first travels down one of two branches depending on the value of x[4] of the persistence representation. The "value" property of each of the two nodes downstream indicates the average binding affinity of the protein-ligand complexes in the training dataset which reached that node, subtracted by the average binding affinity of the entire dataset.

D.2 Full Set of 13 Decision Trees

See Figures O, P, and Q.

E Full benchmarking results

This section shows the performance of PATH and other benchmarked algorithms on predicting binding affinity on PDBBind, BindingDB, and Binding MOAD (Table D), as well as differentiating between active and decoy ligands on the DUD-E dataset (Table E) in numerical form. Because PATH's regression trees can only interpolate, BindingDB and Binding MOAD entries were filtered to only include entries with affinities in the $[-12, 0]$ kcal/mol range (PDBBind's range is $[-8.7, -1.5]$ kcal/mol). Table E shows the AUCs for the protein-ligand complexes for which the various softwares successfully returned a prediction under the following experimental conditions: 1 CPU core, 8GB of memory, and 1 hour of compute time.

F Full List of 77 Features Selected by MDI

Here, we list the top 77 features selected by the highest mean decrease in impurity (MDI) in the 120 initial persistence images constructed in Section B.1. Note that only features

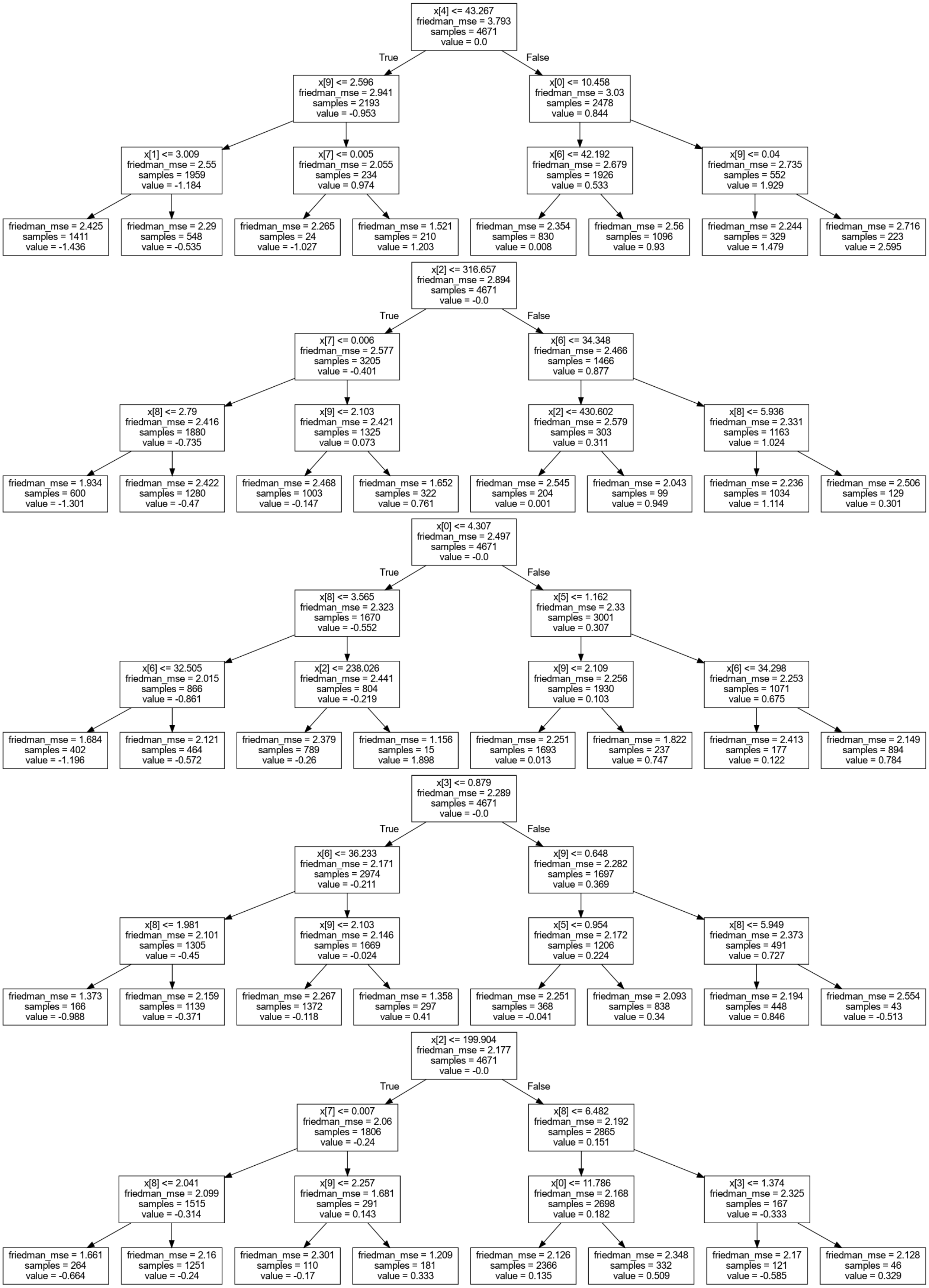

Figure O: Decision trees 1-5 in PATH$^+$

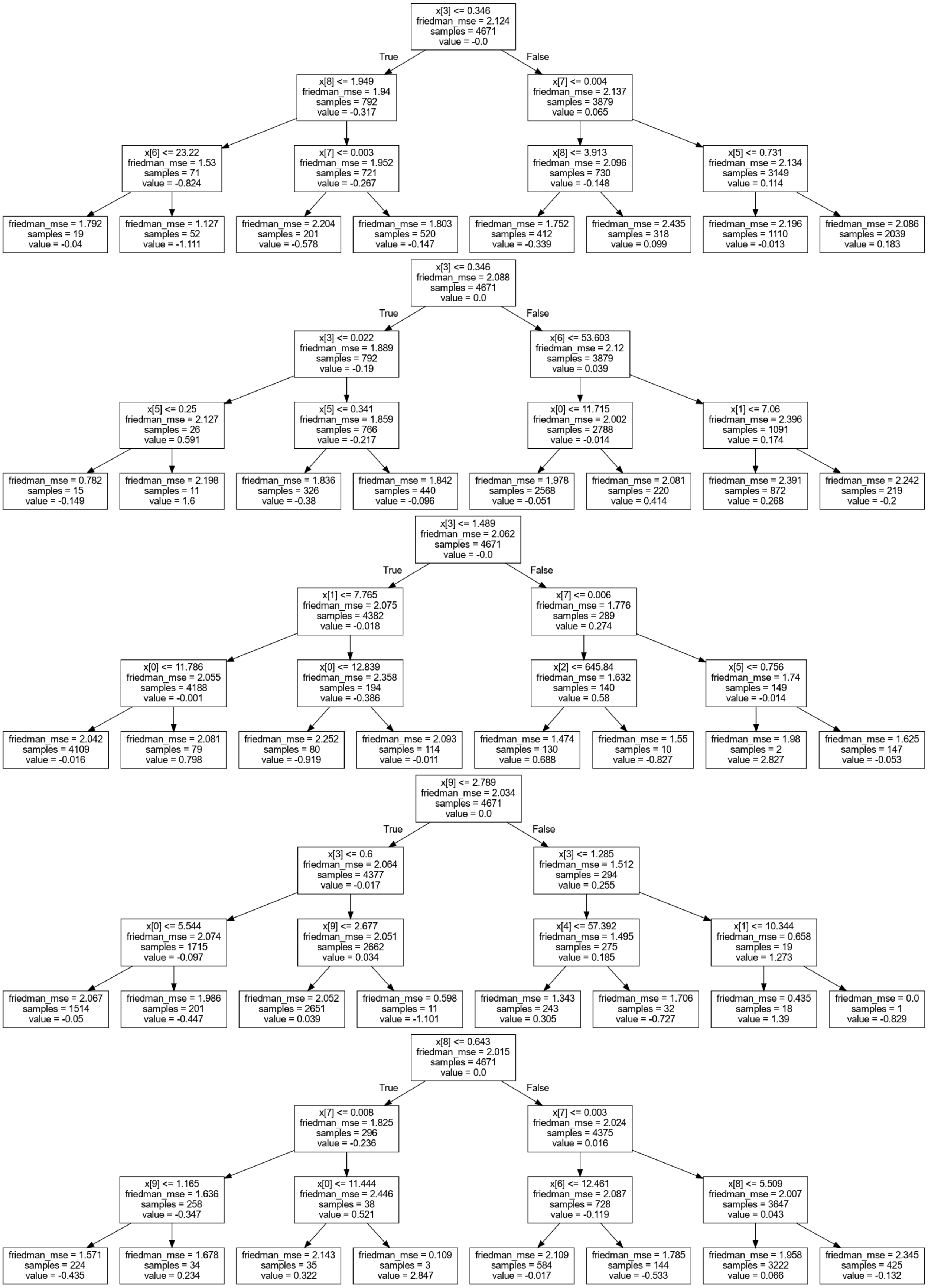

Figure P: Decision trees 6-10 in $PATH^+$

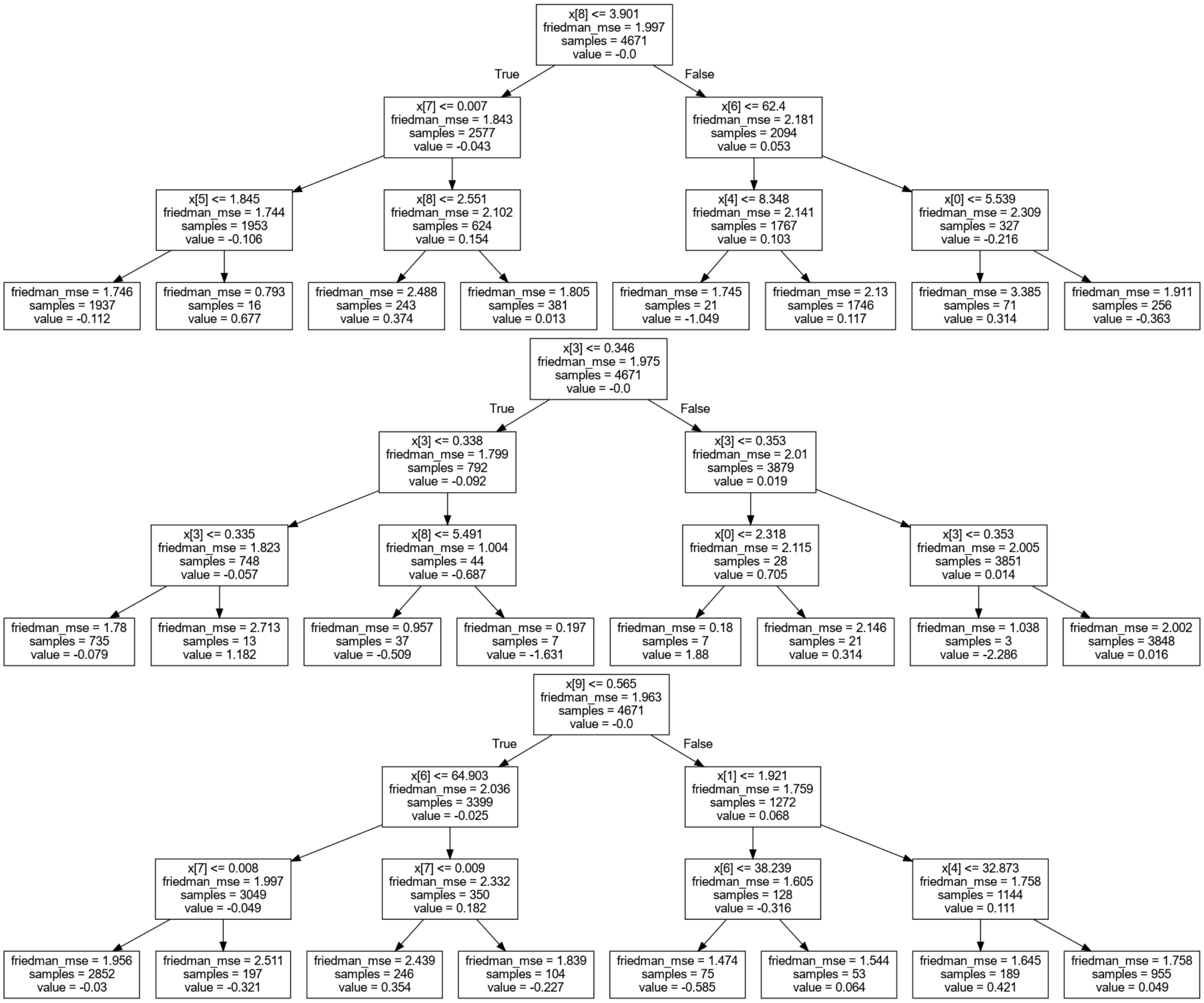

Figure Q: Decision trees 11-13 in PATH^{+}

**Algorithm**	**PDBBind**	**BindingDB**	**Binding MOAD**
AA-Score	4.41	5.15	5.23
ADFR	2.68	3.04	3.05
GNINA	1.9	2.21	2.18
OnionNet	1.53	2.63	2.53
PATH$^+$	2.0	2.24	2.21
PLANET	1.26	2.79	2.63
SMoG2016	2.27	2.41	2.46
TNet-BP	1.68	2.24	2.39
vinardo	2.59	4.15	3.93

Table D: ΔG RMSE (in kcal/mol) of benchmarked binding affinity prediction algorithms on PDBBind, Binding MOAD, and BindingDB.

(i.e., pixels) from persistence images constructed from atom subsets 0-35 with homology dimensions 0 or 1 can be interpreted in the language of IPCs and enjoy the fast compute algorithm we describe in this manuscript. Coincidentally, the top 77 features selected by MDI were all features interpretable in the language of IPCs. In Table F, we report the protein atoms, ligand atoms, IPC dimensions, and distance bin corresponding to the 77 features selected by MDI.

Algorithm	ROC AUC on DUD-E
AA-Score	0.516
AA-Score (Only Finished)	0.514
AutoDock4 (Scoring)	0.525
AutoDock4 (Scoring, Only Finished)	0.543
gnina (Scoring)	0.501
OnionNet	0.513
PLANET	0.514
PLANET (Only Finished)	0.609
SMoG2016	0.514
vinardo (Scoring)	0.510
$PATH^-$	0.696
Vina (Full AD Framework)	Reported 0.69
smina (Full AD Framework)	Reported 0.71
idock (Full AD Framework)	Reported 0.68

Table E: ROC AUCs of binding affinity algorithms on the subset of DUD-E dataset as described in Section 2.4 and Section E. The ROC AUC for Vina, smina, and idock on DUD-E by running the full AutoDock (AD) framework are reported from [75]. "Only finished" indicates that the ROC AUC reported in that row is computed using the protein-ligand complexes for which the row's software successfully returned a prediction, under the experimental conditions described in Section E.

**Protein Atom**	**Ligand Atom**	**IPC Dimension**	**IPC Density Bin (Å)**	**Protein Atom**	**Ligand Atom**	**IPC Dimension**	**IPC Density Bin (Å)**
C	C	1	[4.0, 4.5]	C	N	0	[7.5, 8.0]
C	C	1	[5.0, 5.5]	C	N	0	[8.5, 9.0]
C	C	1	[5.5, 6.0]	C	O	0	[6.5, 7.0]
C	C	1	[6.0, 6.5]	C	S	0	[5.0, 5.5]
C	C	1	[6.5, 7.0]	C	S	0	[9.0, 9.5]
C	C	1	[7.0, 7.5]	C	S	0	[10.5, 11.0]
C	C	1	[7.5, 8.0]	N	C	1	[7.0, 7.5]
C	C	1	[8.0, 8.5]	N	C	1	[7.5, 8.0]
C	C	1	[8.5, 9.0]	N	C	1	[8.0, 8.5]
C	C	1	[9.0, 9.5]	N	C	1	[9.0, 9.5]
C	C	1	[9.5, 10.0]	N	C	1	[9.5, 10.0]
C	C	1	[10.0, 10.5]	N	C	1	[10.0, 10.5]
C	C	1	[10.5, 11.0]	N	C	1	[10.5, 11.0]
C	C	1	[14.5, 15.0]	N	C	1	[11.0, 11.5]
C	C	1	[15.0, 15.5]	N	C	1	[11.5, 12.0]
C	C	1	[15.5, 16.0]	N	C	1	[12.0, 12.5]

Table F: The top 77 features that were selected by MDI. Due to the format of the persistent images, the features in persistent images correspond to IPC features many-to-one manner. Here, we report the deduplicated set of IPC features.

References

[1] Batool M, Ahmad B, Choi S. A structure-based drug discovery paradigm. International journal of molecular sciences. 2019;20(11):2783.

[2] Shoichet BK. Virtual screening of chemical libraries. Nature. 2004;432(7019):862–865.

[3] Kontoyianni M. Docking and virtual screening in drug discovery. Proteomics for drug discovery: Methods and protocols. 2017; p. 255–266.

[4] Maia EHB, Assis LC, De Oliveira TA, Da Silva AM, Taranto AG. Structure-based virtual screening: from classical to artificial intelligence. Frontiers in chemistry. 2020;8:343.

[5] Seo S, Choi J, Park S, Ahn J. Binding affinity prediction for protein–ligand complex using deep attention mechanism based on intermolecular interactions. BMC bioinformatics. 2021;22:1–15.

[6] Li S, Xi L, Wang C, Li J, Lei B, Liu H, et al. A novel method for protein-ligand binding affinity prediction and the related descriptors exploration. Journal of computational chemistry. 2009;30(6):900–909.

[7] Rudicell RS, Kwon YD, Ko SY, Pegu A, Louder MK, Georgiev IS, et al. Enhanced potency of a broadly neutralizing HIV-1 antibody in vitro improves protection against lentiviral infection in vivo. Journal of virology. 2014;88(21):12669–12682.

[8] Zhou M, Li Q, Wang R. Current experimental methods for characterizing protein–protein interactions. ChemMedChem. 2016;11(8):738–756.

[9] Anderson AC. The process of structure-based drug design. Chemistry & biology. 2003;10(9):787–797.

[10] Bash PA, Singh UC, Brown FK, Langridge R, Kollman PA. Calculation of the relative change in binding free energy of a protein-inhibitor complex. Science. 1987;235(4788):574–576.

[11] Aldeghi M, Gapsys V, de Groot BL. Accurate estimation of ligand binding affinity changes upon protein mutation. ACS central science. 2018;4(12):1708–1718.

[12] Mey AS, Allen BK, Macdonald HEB, Chodera JD, Hahn DF, Kuhn M, et al. Best practices for alchemical free energy calculations [article v1.0]. Living journal of computational molecular science. 2020;2(1).

[13] Li Y, Han L, Liu Z, Wang R. Comparative assessment of scoring functions on an updated benchmark: 2. Evaluation methods and general results. Journal of chemical information and modeling. 2014;54(6):1717–1736.

[14] Meli R, Morris GM, Biggin PC. Scoring functions for protein-ligand binding affinity prediction using structure-based deep learning: A review. Frontiers in bioinformatics. 2022;2:57.

[15] Jones D, Kim H, Zhang X, Zemla A, Stevenson G, Bennett WD, et al. Improved protein–ligand binding affinity prediction with structure-based deep fusion inference. Journal of chemical information and modeling. 2021;61(4):1583–1592.

[16] Wang H, Liu H, Ning S, Zeng C, Zhao Y. DLSSAffinity: protein–ligand binding affinity prediction via a deep learning model. Physical Chemistry Chemical Physics. 2022;24(17):10124–10133.

[17] Stepniewska-Dziubinska MM, Zielenkiewicz P, Siedlecki P. Development and evaluation of a deep learning model for protein–ligand binding affinity prediction. Bioinformatics. 2018;34(21):3666–3674.

[18] Jin Z, Wu T, Chen T, Pan D, Wang X, Xie J, et al. CAPLA: improved prediction of protein–ligand binding affinity by a deep learning approach based on a cross-attention mechanism. Bioinformatics. 2023;39(2):btad049.

[19] Yi Y, Wan X, Zhao K, Ou-Yang L, Zhao P. Predicting Protein-Ligand Binding Affinity with Equivariant Line Graph Network. arXiv preprint arXiv:221016098. 2022;.

[20] Li S, Zhou J, Xu T, Huang L, Wang F, Xiong H, et al. Structure-aware interactive graph neural networks for the prediction of protein-ligand binding affinity. In: Proceedings of the 27th ACM SIGKDD Conference on Knowledge Discovery & Data Mining; 2021. p. 975–985.

[21] Pu M, Hayashi T, Cottam H, Mulvaney J, Arkin M, Corr M, et al. Analysis of high-throughput screening assays using cluster enrichment. Statistics in medicine. 2012;31(30):4175–4189.

[22] Rudin C, Chen C, Chen Z, Huang H, Semenova L, Zhong C. Interpretable machine learning: Fundamental principles and 10 grand challenges. Statistic Surveys. 2022;16:1–85.

[23] Jumper J, Evans R, Pritzel A, Green T, Figurnov M, Ronneberger O, et al. Highly accurate protein structure prediction with AlphaFold. Nature. 2021;596(7873):583–589.

[24] Kryshtafovych A, Schwede T, Topf M, Fidelis K, Moult J. Critical assessment of methods of protein structure prediction (CASP)—Round XIV. Proteins: Structure, Function, and Bioinformatics. 2021;89(12):1607–1617.

[25] Buel GR, Walters KJ. Can AlphaFold2 predict the impact of missense mutations on structure? Nature Structural & Molecular Biology. 2022;29(1):1–2.

[26] Pak MA, Markhieva KA, Novikova MS, Petrov DS, Vorobyev IS, Maksimova ES, et al. Using AlphaFold to predict the impact of single mutations on protein stability and function. Plos one. 2023;18(3):e0282689.

[27] Rudin C. Stop explaining black box machine learning models for high stakes decisions and use interpretable models instead. Nature machine intelligence. 2019;1(5):206–215.

[28] Murdoch WJ, Singh C, Kumbier K, Abbasi-Asl R, Yu B. Definitions, methods, and applications in interpretable machine learning. Proceedings of the National Academy of Sciences. 2019;116(44):22071–22080.

[29] Edelsbrunner H, Harer JL. Computational Topology: An Introduction. Hardcover ed. American Mathematical Society; 2009.

[30] Kanari L, Dłotko P, Scolamiero M, Levi R, Shillcock J, Hess K, et al. A topological representation of branching neuronal morphologies. Neuroinformatics. 2018;16:3–13.

[31] Donald BR. Algorithms in structural molecular biology. MIT Press; 2023.

[32] Liang J, Edelsbrunner H, Fu P, Sudhakar PV, Subramaniam S. Analytical shape computation of macromolecules: II. Inaccessible cavities in proteins. Proteins: Structure, Function, and Bioinformatics. 1998;33(1):18–29.

[33] Cang Z, Wei GW. Integration of element specific persistent homology and machine learning for protein-ligand binding affinity prediction. International journal for numerical methods in biomedical engineering. 2018;34(2):e2914.

[34] Cang Z, Mu L, Wei GW. Representability of algebraic topology for biomolecules in machine learning based scoring and virtual screening. PLoS computational biology. 2018;14(1):e1005929.

[35] Wu K, Zhao Z, Wang R, Wei GW. TopP–S: Persistent homology-based multi-task deep neural networks for simultaneous predictions of partition coefficient and aqueous solubility. Journal of computational chemistry. 2018;39(20):1444–1454.

[36] Wang M, Cang Z, Wei GW. A topology-based network tree for the prediction of protein–protein binding affinity changes following mutation. Nature Machine Intelligence. 2020;2(2):116–123.

[37] Wee J, Xia K. Persistent spectral based ensemble learning (PerSpect-EL) for protein–protein binding affinity prediction. Briefings in Bioinformatics. 2022;23(2):bbac024.

[38] Liu X, Feng H, Wu J, Xia K. Dowker complex based machine learning (DCML) models for protein-ligand binding affinity prediction. PLoS Computational Biology. 2022;18(4):e1009943.

[39] Nguyen DD, Cang Z, Wu K, Wang M, Cao Y, Wei GW. Mathematical deep learning for pose and binding affinity prediction and ranking in D3R Grand Challenges. Journal of computer-aided molecular design. 2019;33:71–82.

[40] Gaieb Z, Parks CD, Chiu M, Yang H, Shao C, Walters WP, et al. D3R Grand Challenge 3: blind prediction of protein–ligand poses and affinity rankings. Journal of computer-aided molecular design. 2019;33:1–18.

[41] Cohen-Steiner D, Edelsbrunner H, Harer J. Stability of persistence diagrams. In: Proceedings of the twenty-first annual symposium on Computational geometry; 2005. p. 263–271.

[42] DePristo MA, de Bakker PI, Blundell TL. Heterogeneity and inaccuracy in protein structures solved by X-ray crystallography. Structure. 2004;12(5):831–838.

[43] Cang Z, Wei GW. TopologyNet: Topology based deep convolutional and multi-task neural networks for biomolecular property predictions. PLoS computational biology. 2017;13(7):e1005690.

[44] Hu L, Benson ML, Smith RD, Lerner MG, Carlson HA. Binding MOAD (mother of all databases). Proteins: Structure, Function, and Bioinformatics. 2005;60(3):333–340.

[45] Ahmed A, Smith RD, Clark JJ, Dunbar Jr JB, Carlson HA. Recent improvements to Binding MOAD: a resource for protein–ligand binding affinities and structures. Nucleic acids research. 2015;43(D1):D465–D469.

[46] Smith RD, Clark JJ, Ahmed A, Orban ZJ, Dunbar Jr JB, Carlson HA. Updates to binding MOAD (mother of all databases): polypharmacology tools and their utility in drug repurposing. Journal of molecular biology. 2019;431(13):2423–2433.

[47] Wagle S, Smith RD, Dominic III AJ, DasGupta D, Tripathi SK, Carlson HA. Sunsetting Binding MOAD with its last data update and the addition of 3D-ligand polypharmacology tools. Scientific Reports. 2023;13(1):3008.

[48] Gilson MK, Liu T, Baitaluk M, Nicola G, Hwang L, Chong J. BindingDB in 2015: a public database for medicinal chemistry, computational chemistry and systems pharmacology. Nucleic acids research. 2016;44(D1):D1045–D1053.

[49] Liu T, Lin Y, Wen X, Jorissen RN, Gilson MK. BindingDB: a web-accessible database of experimentally determined protein–ligand binding affinities. Nucleic acids research. 2007;35(suppl_1):D198–D201.

[50] Wang R, Fang X, Lu Y, Wang S. The PDBbind database: Collection of binding affinities for protein- ligand complexes with known three-dimensional structures. Journal of medicinal chemistry. 2004;47(12):2977–2980.

[51] Liu Z, Su M, Han L, Liu J, Yang Q, Li Y, et al. Forging the basis for developing protein–ligand interaction scoring functions. Accounts of chemical research. 2017;50(2):302–309.

[52] Wang Y, Huang H, Rudin C, Shaposhnik Y. Understanding How Dimension Reduction Tools Work: An Empirical Approach to Deciphering t-SNE, UMAP, TriMap, and PaCMAP for Data Visualization. Journal of Machine Learning Research. 2021;22(201):1–73.

[53] Yang J, Roy A, Zhang Y. BioLiP: a semi-manually curated database for biologically relevant ligand–protein interactions. Nucleic acids research. 2012;41(D1):D1096–D1103.

[54] Liu X, Feng H, Wu J, Xia K. Hom-complex-based machine learning (HCML) for the prediction of protein–protein binding affinity changes upon mutation. Journal of chemical information and modeling. 2022;62(17):3961–3969.

[55] Liu X, Feng H, Lü Z, Xia K. Persistent Tor-algebra for protein–protein interaction analysis. Briefings in Bioinformatics. 2023;24(2):bbad046.

[56] Liu X, Wang X, Wu J, Xia K. Hypergraph-based persistent cohomology (HPC) for molecular representations in drug design. Briefings in Bioinformatics. 2021;22(5):bbaa411.

[57] Chen CY, Georgiev I, Anderson AC, Donald BR. Computational structure-based redesign of enzyme activity. Proceedings of the National Academy of Sciences. 2009;106(10):3764–3769.

[58] Do Kwon Y, Pancera M, Acharya P, Georgiev IS, Crooks ET, Gorman J, et al. Crystal structure, conformational fixation and entry-related interactions of mature ligand-free HIV-1 Env. Nature structural & molecular biology. 2015;22(7):522–531.

[59] Holt GT, Gorman J, Wang S, Lowegard AU, Zhang B, Liu T, et al. Improved HIV-1 neutralization breadth and potency of V2-apex antibodies by in silico design. Cell reports. 2023;42(7).

[60] Quinlan JR. Induction of decision trees. Machine learning. 1986;1:81–106.

[61] Louppe G. Understanding random forests: From theory to practice. arXiv preprint arXiv:14077502. 2014;.

[62] Zhang R, Xin R, Seltzer M, Rudin C. Optimal Sparse Regression Trees. In: Proceedings of the AAAI Conference on Artificial Intelligence. vol. 37; 2023. p. 11270–11279.

[63] Xin R, Zhong C, Chen Z, Takagi T, Seltzer M, Rudin C. Exploring the whole rashomon set of sparse decision trees. Advances in Neural Information Processing Systems. 2022;35:14071–14084.

[64] Fan C, Liu D, Huang R, Chen Z, Deng L. PredRSA: a gradient boosted regression trees approach for predicting protein solvent accessibility. In: Bmc Bioinformatics. vol. 17. BioMed Central; 2016. p. 85–95.

[65] Zhou C, Yu H, Ding Y, Guo F, Gong XJ. Multi-scale encoding of amino acid sequences for predicting protein interactions using gradient boosting decision tree. PLoS One. 2017;12(8):e0181426.

[66] Deng L, Sui Y, Zhang J. XGBPRH: prediction of binding hot spots at protein–RNA interfaces utilizing extreme gradient boosting. Genes. 2019;10(3):242.

[67] Hallen MA, Martin JW, Ojewole A, Jou JD, Lowegard AU, Frenkel MS, et al. OSPREY 3.0: open-source protein redesign for you, with powerful new features. Journal of computational chemistry. 2018;39(30):2494–2507.

[68] Ravindranath PA, Forli S, Goodsell DS, Olson AJ, Sanner MF. AutoDockFR: advances in protein-ligand docking with explicitly specified binding site flexibility. PLoS computational biology. 2015;11(12):e1004586.

[69] Quiroga R, Villarreal MA. Vinardo: A scoring function based on autodock vina improves scoring, docking, and virtual screening. PloS one. 2016;11(5):e0155183.

[70] McNutt AT, Francoeur P, Aggarwal R, Masuda T, Meli R, Ragoza M, et al. GNINA 1.0: molecular docking with deep learning. Journal of cheminformatics. 2021;13(1):43.

[71] Pan X, Wang H, Zhang Y, Wang X, Li C, Ji C, et al. AA-score: a new scoring function based on amino acid-specific interaction for molecular docking. Journal of Chemical Information and Modeling. 2022;62(10):2499–2509.

[72] Debroise T, Shakhnovich EI, Chéron N. A hybrid knowledge-based and empirical scoring function for protein–ligand interaction: SMoG2016. Journal of chemical information and modeling. 2017;57(3):584–593.

[73] Zheng L, Fan J, Mu Y. OnionNet: a multiple-layer intermolecular-contact-based convolutional neural network for protein–ligand binding affinity prediction. ACS omega. 2019;4(14):15956–15965.

[74] Zhang X, Gao H, Wang H, Chen Z, Zhang Z, Chen X, et al. PLANET: a multi-objective graph neural network model for protein–ligand binding affinity prediction. Journal of Chemical Information and Modeling. 2023;64(7):2205–2220.

[75] Masters L, Eagon S, Heying M. Evaluation of consensus scoring methods for AutoDock Vina, smina and idock. Journal of Molecular Graphics and Modelling. 2020;96:107532.

[76] David V, Grinberg N, Moldoveanu SC, Grinberg N, Moldoveanu S. Long-range molecular interactions involved in the retention mechanisms of liquid chromatography. Advances in chromatography. 2017; p. 73–110.

[77] Morris GM, Huey R, Lindstrom W, Sanner MF, Belew RK, Goodsell DS, et al. AutoDock4 and AutoDockTools4: Automated docking with selective receptor flexibility. Journal of computational chemistry. 2009;30(16):2785–2791.

[78] Eberhardt J, Santos-Martins D, Tillack AF, Forli S. AutoDock Vina 1.2. 0: New docking methods, expanded force field, and python bindings. Journal of chemical information and modeling. 2021;61(8):3891–3898.

[79] Koes DR, Baumgartner MP, Camacho CJ. Lessons learned in empirical scoring with smina from the CSAR 2011 benchmarking exercise. Journal of chemical information and modeling. 2013;53(8):1893–1904.

[80] Li H, Leung KS, Wong MH. idock: A multithreaded virtual screening tool for flexible ligand docking. In: 2012 IEEE Symposium on Computational Intelligence in Bioinformatics and Computational Biology (CIBCB). IEEE; 2012. p. 77–84.

[81] Liu F, Kovalevsky AY, Tie Y, Ghosh AK, Harrison RW, Weber IT. Effect of flap mutations on structure of HIV-1 protease and inhibition by saquinavir and darunavir. Journal of molecular biology. 2008;381(1):102–115.

[82] Kovalevsky AY, Tie Y, Liu F, Boross PI, Wang YF, Leshchenko S, et al. Effectiveness of nonpeptide clinical inhibitor TMC-114 on HIV-1 protease with highly drug resistant mutations D30N, I50V, and L90M. Journal of medicinal chemistry. 2006;49(4):1379–1387.

[83] Pinard MA, Boone CD, Rife BD, Supuran CT, McKenna R. Structural study of interaction between brinzolamide and dorzolamide inhibition of human carbonic anhydrases. Bioorganic & Medicinal Chemistry. 2013;21(22):7210–7215.

[84] Li S, Wan F, Shu H, Jiang T, Zhao D, Zeng J. MONN: a multi-objective neural network for predicting compound-protein interactions and affinities. Cell systems. 2020;10(4):308–322.

[85] Yan J, Ye Z, Yang Z, Lu C, Zhang S, Liu Q, et al. Multi-task bioassay pre-training for protein-ligand binding affinity prediction. Briefings in Bioinformatics. 2024;25(1):bbad451.

[86] Hu F, Jiang J, Yin P. Interpretable prediction of protein-ligand interaction by convolutional neural network. In: 2019 IEEE International Conference on Bioinformatics and Biomedicine (BIBM). IEEE; 2019. p. 656–659.

[87] Luo D, Liu D, Qu X, Dong L, Wang B. Enhancing generalizability in protein–ligand binding affinity prediction with multimodal contrastive learning. Journal of Chemical Information and Modeling. 2024;64(6):1892–1906.

[88] Wu MH, Xie Z, Zhi D. A Folding-Docking-Affinity framework for protein-ligand binding affinity prediction. Communications Chemistry. 2025;8(1):1–9.

[89] Simonyan K, Vedaldi A, Zisserman A. Deep inside convolutional networks: Visualising image classification models and saliency maps. arXiv preprint arXiv:13126034. 2013;.

[90] Elhage N, Nanda N, Olsson C, Henighan T, Joseph N, Mann B, et al. Superposition, memorization, and double descent. Transformer Circuits Thread. 2022;.

[91] Bricken T, Templeton A, Batson J, Chen B, Jermyn A, Conerly T, et al. Decomposing Language Models With Dictionary Learning. Transformer Circuits Thread. 2023;.

[92] Ameisen E, Lindsey J, Pearce A, Gurnee W, Turner NL, Chen B, et al. Circuit tracing: Revealing computational graphs in language models. Transformer Circuits Thread. 2025;.

[93] Bills S, Cammarata N, Mossing D, Tillman H, Gao L, Goh G, et al. Language models can explain neurons in language models. OpenAI Blog. 2023;2.

[94] Gorczynski MJ, Grembecka J, Zhou Y, Kong Y, Roudaia L, Douvas MG, et al. Allosteric inhibition of the protein-protein interaction between the leukemia-associated proteins Runx1 and CBFβ. Chemistry & biology. 2007;14(10):1186–1197.

[95] Qi Y, Martin JW, Barb AW, Thélot F, Yan AK, Donald BR, et al. Continuous interdomain orientation distributions reveal components of binding thermodynamics. Journal of molecular biology. 2018;430(18):3412–3426.

[96] Wang S, Reeve SM, Holt GT, Ojewole AA, Frenkel MS, Gainza P, et al. Chiral evasion and stereospecific antifolate resistance in Staphylococcus aureus. PLoS Computational Biology. 2022;18(2):e1009855.

[97] Wang DD, Chan MT. Protein-ligand binding affinity prediction based on profiles of intermolecular contacts. Computational and Structural Biotechnology Journal. 2022;20:1088–1096.

[98] Wójcikowski M, Kukiełka M, Stepniewska-Dziubinska MM, Siedlecki P. Development of a protein–ligand extended connectivity (PLEC) fingerprint and its application for binding affinity predictions. Bioinformatics. 2019;35(8):1334–1341.

[99] Sheehy DR. Linear-size approximations to the Vietoris-Rips filtration. In: Proceedings of the twenty-eighth annual symposium on Computational geometry; 2012. p. 239–248.

[100] Choudhary A, Kerber M, Raghvendra S. Improved approximate rips filtrations with shifted integer lattices and cubical complexes. Journal of Applied and Computational Topology. 2021;5(3):425–458.

[101] Čufar M, Virk Ž. Fast computation of persistent homology representatives with involuted persistent homology. arXiv preprint arXiv:210503629. 2021;.

[102] Ojewole A, Lowegard A, Gainza P, Reeve SM, Georgiev I, Anderson AC, et al. OSPREY predicts resistance mutations using positive and negative computational protein design. Computational Protein Design. 2017; p. 291–306.

[103] Huynh K, Kibrom A, Donald BR, Zhou P. Discovery, characterization, and redesign of potent antimicrobial thanatin orthologs from Chinavia ubica and Murgantia histrionica targeting E. coli LptA. Journal of Structural Biology: X. 2023;8:100091. doi:https://doi.org/10.1016/j.yjsbx.2023.100091.

[104] Fasy BT, Patel A. Persistent Homology Transform Cosheaf. arXiv preprint arXiv:220805243. 2022;.

[105] consortium w. Protein Data Bank: the single global archive for 3D macromolecular structure data. Nucleic Acids Research. 2018;47(D1):D520–D528. doi:10.1093/nar/gky949.

[106] Zomorodian A, Carlsson G. Computing persistent homology. In: Proceedings of the twentieth annual symposium on Computational geometry; 2004. p. 347–356.

[107] Anand DV, Meng Z, Xia K, Mu Y. Weighted persistent homology for osmolyte molecular aggregation and hydrogen-bonding network analysis. Scientific reports. 2020;10(1):9685.

[108] Adams H, Emerson T, Kirby M, Neville R, Peterson C, Shipman P, et al. Persistence images: A stable vector representation of persistent homology. Journal of Machine Learning Research. 2017;18.

[109] Liu Z, Li Y, Han L, Li J, Liu J, Zhao Z, et al. PDB-wide collection of binding data: current status of the PDBbind database. Bioinformatics. 2015;31(3):405–412.

[110] Vega S, Kang LW, Velazquez-Campoy A, Kiso Y, Amzel LM, Freire E. A structural and thermodynamic escape mechanism from a drug resistant mutation of the HIV-1 protease. Proteins: Structure, Function, and Bioinformatics. 2004;55(3):594–602.

[111] Pedregosa F, Varoquaux G, Gramfort A, Michel V, Thirion B, Grisel O, et al. Scikit-learn: Machine Learning in Python. Journal of Machine Learning Research. 2011;12:2825–2830.

[112] Menze BH, Kelm BM, Masuch R, Himmelreich U, Bachert P, Petrich W, et al. A comparison of random forest and its Gini importance with standard chemometric methods for the feature selection and classification of spectral data. BMC bioinformatics. 2009;10:1–16.

[113] Merrick L. Randomized ablation feature importance. arXiv preprint arXiv:191000174. 2019;.

[114] Le Gall F. Powers of tensors and fast matrix multiplication. In: Proceedings of the 39th international symposium on symbolic and algebraic computation; 2014. p. 296–303.

[115] Kim E, Baker C, Dwyer M, Murcko M, Rao B, Tung R, et al. Crystal structure of HIV-1 protease in complex with VX-478, a potent and orally bioavailable inhibitor of the enzyme. Journal of the American Chemical Society. 1995;117(3):1181–1182.

[116] Scott DE, Ehebauer MT, Pukala T, Marsh M, Blundell TL, Venkitaraman AR, et al. Using a fragment-based approach to target protein–protein interactions. ChemBioChem. 2013;14(3):332–342.

[117] Westbrook JD, Shao C, Feng Z, Zhuravleva M, Velankar S, Young J. The chemical component dictionary: complete descriptions of constituent molecules in experimentally determined 3D macromolecules in the Protein Data Bank. Bioinformatics. 2015;31(8):1274–1278.

[118] Pandala SR. LazyPredict; 2022. `https://github.com/shankarpandala/lazypredict`.

[119] Edelsbrunner H, Harer J, et al. Persistent homology-a survey. Contemporary mathematics. 2008;453(26):257–282.

[120] Fugacci U, Scaramuccia S, Iuricich F, De Floriani L, et al. Persistent Homology: a Step-by-step Introduction for Newcomers. In: STAG; 2016. p. 1–10.

[121] Hatcher A. Algebraic Topology. Cambridge, England: Cambridge University Press; 2001.

[122] Adams H, Segert J. Simplicial complex filtration demonstrations in Mathematica;. Available from: `https://www.math.colostate.edu/~adams/research/`.

[123] Maria C, Boissonnat JD, Glisse M, Yvinec M. The GUDHI library: Simplicial complexes and persistent homology. In: Mathematical Software–ICMS 2014: 4th International Congress, Seoul, South Korea, August 5-9, 2014. Proceedings 4. Springer; 2014. p. 167–174.

[124] Tauzin G, Lupo U, Tunstall L, Pérez JB, Caorsi M, Medina-Mardones AM, et al. giotto-tda: A topological data analysis toolkit for machine learning and data exploration. The Journal of Machine Learning Research. 2021;22(1):1834–1839.

[125] Pérez JB, Hauke S, Lupo U, Caorsi M, Dassatti A. giotto-ph: A Python Library for High-Performance Computation of Persistent Homology of Vietoris–Rips Filtrations; 2021.

[126] Bauer U. Ripser: efficient computation of Vietoris-Rips persistence barcodes. J Appl Comput Topol. 2021;5(3):391–423. doi:10.1007/s41468-021-00071-5.

[127] Chazal F, Cohen-Steiner D, Glisse M, Guibas LJ, Oudot SY. Proximity of persistence modules and their diagrams. In: Proceedings of the twenty-fifth annual symposium on Computational geometry; 2009. p. 237–246.

[128] Chazal F, De Silva V, Oudot S. Persistence stability for geometric complexes. Geometriae Dedicata. 2014;173(1):193–214.

[129] Dlotko P. Persistence representations. In: GUDHI User and Reference Manual. GUDHI Editorial Board; 2017.

[130] Friedman JH. Greedy function approximation: a gradient boosting machine. Annals of statistics. 2001; p. 1189–1232.

[131] Zhang C, Zhang X, Freddolino PL, Zhang Y. BioLiP2: an updated structure database for biologically relevant ligand–protein interactions. Nucleic Acids Research. 2023; p. gkad630.

[132] McInnes L, Healy J, Melville J. UMAP: Uniform manifold approximation and projection for dimension reduction. arXiv preprint arXiv:180203426. 2018;.

[133] Van der Maaten L, Hinton G. Visualizing data using t-SNE. Journal of machine learning research. 2008;9(11).

[134] Boissonnat JD, Pritam S. Computing persistent homology of flag complexes via strong collapses. 35th International Symposium on Computational Geometry. 2018;.

[135] Milosavljević N, Morozov D, Skraba P. Zigzag persistent homology in matrix multiplication time. In: Proceedings of the twenty-seventh Annual Symposium on Computational Geometry; 2011. p. 216–225.

[136] Hales TC. A proof of the Kepler conjecture. Annals of mathematics. 2005; p. 1065–1185.
