# Supplementary material for: Predicting Affinity Through Homology (PATH): Interpretable binding affinity prediction with persistent homology
Source: PLoS Comput Biol. 2025 Jun 27;21(6):e1013216. doi: 10.1371/journal.pcbi.1013216 (PMC12226026; doi:10.1371/journal.pcbi.1013216)
Supplement: Attachment — Submitted filename: response-to-reviewers.pdf [file pcbi.1013216.s002.pdf]

How We Responded to Reviewers' Comments: Predicting Affinity Through Homology (PATH): Interpretable Binding Affinity Prediction with Persistent Homology

Yuxi Long, Bruce R. Donald

General Response

We thank the editors' nice assessment of our paper and for requesting a minor revision. We thank the reviewers for helpful comments, which have greatly improved our manuscript. Below, we address each comment individually and summarize the resulting changes to the manuscript. While changes to the manuscript have been made throughout, the major ones are indicated in red. Added bibliographic references are not in red in the manuscript due to limitations of LaTeX.

Editor Comments

> This is a very interesting method for predicting binding affinities. Both the reviewers and we are enthusiastic about this work. Please address the reviewer comments in your revised version.
>
> Congratulations on a very nice contribution.

Answer:

Thank you very much for your summary of our paper, and for your enthusiasm. We have addressed the reviewers' comments below.

Reviewer 1

The authors present the development of a machine learning approach to predict binding affinities for molecular complexes based on their three-dimensional structures. The main algorithm is called PATH+, and its strength is not increased accuracy beyond existing methods, but rather increased generalizability across datasets and a potential for greater interpretability at comparable or better accuracy. PATH+ also has reduced computational complexity compared to other methods. The gains in these dimensions beyond accuracy are noteworthy, and it is important that algorithmic development in the field progress across all of these dimensions, and others as well. PATH+ makes use of advances in computational topology, including persistent homology, and develops useful persistence fingerprints. The authors also present a scoring algorithm PATH-, that distinguishes binders from non-binders. The paper is reasonably well written and reasonably clear. The suggestions below are made for the authors to consider and incorporate at their own discretion.

Answer:

We thank the reviewer for positive feedback and the thoroughly detailed review. We also thank the reviewer for letting us incorporate comments at our own discretion.

(1) The standard sections of the paper are exceedingly short. By the end of page 12, one has completed abstract, author summary, introduction, results, and discussion. While some results are presented, much is only alluded to and a great deal is claimed but not shown at the level necessary to make a convincing case to the reader. As but one example, we are told that PATH+ reduces the 14,472 features of a previous method, TNet-BP, which also predicts binding affinity using persistent homology, to only 10 features in PATH+, but there is no real discussion of the differences and what insights we can gather from those differences. Too much of the paper is buried in the overly long methods section.

Answer:

Thank you for these comments and suggestions. We made the following changes:

1. We moved the table containing the persistence fingerprint features to the Results section.
2. We extended Sec. 3 Discussion to also highlight that part of our contribution is that we explored the potential of persistence homology features for creating great algorithms.
3. We made more explicit what we found in the drastic reduction of features from TNet-BP in Sec. 3 Discussion: Specifically, we discovered that many features used for TNet-BP are unnecessary and it is also unnecessary to use a neural network to analyze these features. We further added SI Section F containing an extended set of important features that are selected by mean decrease in impurity after fitting an initial ensemble of gradient boosted trees to a superset of TNet-BP features. Remarkably, all 77 selected features by MDI are interpretable in the language of IPCs, even though many features in TNet-BP do not under the language of IPCs and thus additionally do not enjoy the fast algorithm for computation that we present in this work.

(2) The claim is made that PATH+ is fully interpretable, but the point is made by argument and not effectively by illustration. We are told this is true because persistence fingerprints are readily interpretable in terms of atomic interactions, and that the decision-tree structure inherent in PATH+ leads to decision-tree-like constructs on atomic interactions, which are therefore interpretable (it reads like a proof). But the real question is whether the interpretations that result from machine learning correspond to those of structure biophysical chemistry, or whether they are something different? And if different, are they at least as useful? Two examples from the text, HIV and CA II, read much more like rationalizations than actual explanations that come from a complete analysis of the ML result. Working out examples in much more detail and much more critically than has been done here, would more meaningfully support the point that the interpretability of PATH+ can lead to trust, which otherwise remains to be seen.

Answer:

We examined the carbonic anhydrase II complex more closely (changed Fig. 7) and further developed our hypothesis in Sec. 3 (Discussion) about the way that persistence fingerprint is potentially capturing the biophysical interactions of a protein-ligand complex. Namely, we believe persistence fingerprint may capture binding activity by considering the location of protein backbone atoms at a distance such that their attached side chains would make favorable interactions. We hypothesize that, compared to physics-based methods that consider atomic interactions in the static structure, the location of backbone atoms and their corresponding opposition distances capture ensemble averages that are normally only available through molecular dynamics, thus making persistence fingerprint a more robust indicator of binding activity.

(3) The exact problem statement may be somewhere in the paper, but this reader didn't find it clearly stated at the start. Are unbound structures the input, bound structures, or both? The answer appears to be bound structures, but is this a requirement? Much has been written about conformational changes on binding and the effects that this can have on binding energy. Does the use of bound structures limit the accuracy that can be achieved?

Answer:

Thank you, we have now revised to address this question. PATH takes in bound protein-ligand structures, likely generated by docking softwares. We now point out the input and output of PATH in the very first paragraph of Sec. 1 Introduction.

> (4) The authors repeated state that the persistence fingerprint encodes information about topology at different scales, but what about other biophysical features thought to be important for binding? What is encoded in a persistence fingerprint, what additional information could be added by learning over the data, and how does this correspond to what a physics-based modeling approach could capture? Conformational energy changes upon binding? Conformational entropy losses upon binding? Desolvation penalties? The hydrophobic effect? Hydrogen bonds? Quantum-mechanical effects? pH dependence of binding due to titration changes? Temperature dependence? How dependent is the algorithm on knowing accurate titration states? It appears that PATH+ uses a set of concepts that are not directly related to these biophysical concepts but that are none-the-less useful. The extent to which they can be related to biophysical explanations could be interesting to discuss, and might lead to suggestions for additional features for future investigation. The presentation here seems to claim too much and give short shrift to what are truly good questions.

Answer:

Indeed, persistence fingerprint doesn't directly encode biophysical information. Based on the protein atoms, ligand atoms, and their distances that persistence fingerprint corresponds to, we hypothesize that persistence fingerprint captures an ensemble average of the protein-ligand conformation. We added a discussion on this in the extended Sec. 3 Discussion for a potential future investigation.

> (5) The last sentence of the first paragraph should be reconsidered ("A reliable ranking of docking poses, based on affinity, potency, or other biophysical properties, is essential for accurate SBDD [69].") The authors don't seem to believe this based on their introduction of PATH-; they seem to realize that separating the vast majority of non-binders and weak binders from strong binders is valuable, and some reasonable ranking of strong binders is even better. But certainly correct ranking of the weak binders is not at all necessary. I point this out because the topic of the paper is important, and there are many places where a thoughtful argument is necessary to present important subtleties.

Answer:

Thank you for the suggestion. We added sentences that highlight the importance of separating binding vs non-binding poses in the beginning paragraphs 1 and 2 of Sec. 1 and in the last paragraph of Sec. 3.

The more subtle point is this: We find there are three important facets for drug design, all of which are important for designing chemically active compounds:

1. Discriminating binders vs non-binders. This narrows down the molecule search space for the other two facets and is what PATH^{-} seeks to address.
2. Predicting the approximate strength of binders, as measured by Pearson correlation. This allows for selecting strong binders, which are likely to be more chemically active. This is what PATH^{+} seeks to address.
3. Ranking the strength of binders, as measured by Spearman correlation. This allows further optimization of strong binders. This is well addressed by OSPREY [1] and other protein

> design tools that model local interactions.
>
> Note that while a perfect Pearson correlation would imply a perfect Spearman correlation, an algorithm with an imperfect but high Pearson correlation might not have high Spearman correlation. For example, because regression trees can only output a finite number of values, $PATH^+$ will predict the same affinity for protein-ligand complexes with similar affinities. The last paragraph of Sec. 3 ("A current weakness of $PATH^+$ ...") discusses this limitation and and suggests a user to resort to protein design tools to rank compounds upon small mutations.

The protein design tool from our lab, OSPREY, was developed and improved over 20 years and it has been experimentally validated many times that OSPREY very well approximates the binding affinity landascape locally – in other words, OSPREY produces very good ranking of protein-ligand complexes that are similar in structure [2, 3, 4]. $PATH^+$ and $PATH^-$ seek to complement OSPREY by providing interpretable and generalizable approaches to the other steps above in the drug discovery process. We hope the revised first two paragraphs of Sec. 1 and the last paragraph of Sec. 3 will point the reader to this subtlety.

> (6) In the last sentence of the first paragraph of section 2.1, the word "predictions" should be "prediction"

Answer:

Thank you for your attentiveness. This is now fixed.

> (7) The opposition distance is not defined until the methods section, although it is used earlier. It would be simple enough to say what it was when first used.

Answer:

Done. The opposition distance is first used in Results paragraph 1, and it is defined there now.

Reviewer 2

> The study introduces PATH (Predicting Affinity Through Homology), an interpretable algorithm for predicting protein-ligand binding affinity. It uses a new method to compute persistent homology features for protein-ligand complexes that is more computationally efficient than previous methods and independent of protein size. The algorithm uses inter-nuclear persistent contours (IPCs) and persistence fingerprints to represent protein-ligand interactions. The SI details the persistent homology, construction of IPCs, and feature selection along with the benchmarking of PATH against previous binding affinity prediction algorithms.
>
> Overall, this manuscript is clear, concise, and well-written. The research topic is both interesting and relevant to PLOS Computational Biology. The results indicate that PATH performs comparably to other state-of-the-art methods while exhibiting less overfitting, and it also allows for interpretability. The source code for PATH is available in a GitHub repository. Below are some specific concerns:

Answer:

We thank the reviewer for the detailed review and enthusiasm about our work.

> The persistence fingerprint used in the PATH algorithm consists of 10 features. While this reduction in dimensionality is presented as an advantage, does increasing the number of features to around 20, where the performance of trained GBRs saturates (see Fig. 14), enhance overall performance? Additionally, how does the computational time increase with the addition of more features?

Answer:

Thank you for the suggestion. To address this comment, we performed additional experiments on constructing models with 5, 10, 20, 40 or 70 features and added their results in SI Sec. B.3.1. We find that:

- Indeed, there is a slight performance gain when using more features (measured by RMSE on orthogonal datasets).
- Coincidentally, all of the top 77 features selected by MDI are can be interpreted in the language of IPCs (SI Sec. F). Therefore, using any of these 77 features will enjoy the fast and provably accurate compute algorithm that we propose in this work.

We see that a user might want to use an alterative number of features for better accuracy or interpretability. Therefore, we fully include our findings in SI Sections B.3.1 and F.

> While the authors demonstrate the algorithm's performance on various datasets, such as PDBBind and BindingDB, it remains unclear how well the method generalizes to different protein families and ligand types.

Answer:

Thank you. We added SI Sec. B.6, which shows the average performance of PATH+ on different ligand types as annotated by the PDB Chemical Component Dictionary [5]. PATH+ shows different level of performance for different ligand types

The same figure is attached here (Figure 1).

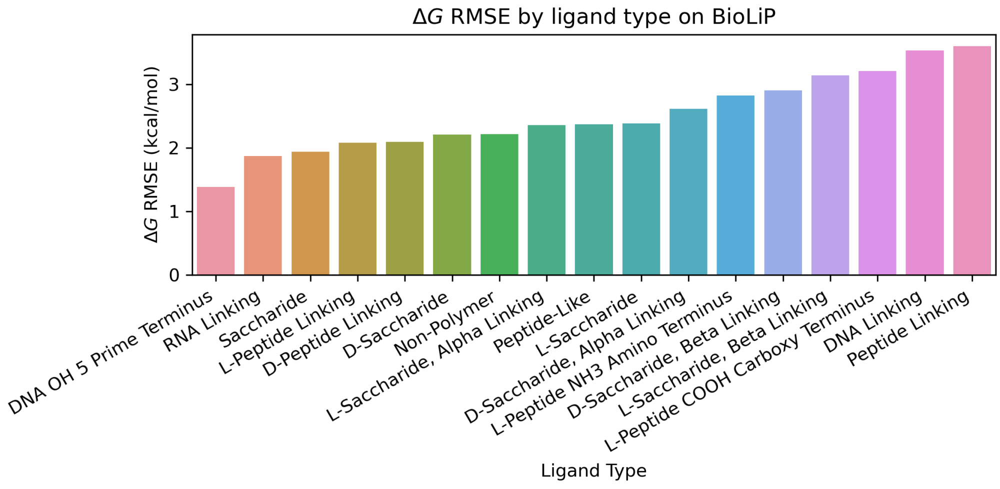

Figure 1: **The performance of PATH+ across different ligand types.** Shown: Root mean squared error of PATH+'s predicted ΔG (kcal/mol) across different ligand types in BioLiP.

> The authors provided a detailed comparison of PATH with other state-of-the-art approaches, but I did not find the algorithm's relative strengths and weaknesses compared to other deep learning-based affinity prediction methods.

Answer:

Yes. Compared to deep learning approaches,

+ PATH is far more interpretable, even compared to the explainability that recent deep learning algorithms incorporate.
+ PATH is more generalizable, empirically measured.
− PATH struggles to rank protein-ligand complexes with small affinity differences. This can be remedied in future work.

We added an extended discussion about these things in Sec. 3 Discussion.

> In SI: Fig. 14, the authors presented RMSE versus the number of remaining features, identifying 10 features as the persistence fingerprint with 100 estimators (trees). How these RMSEs vary with 13 estimators as mentioned in Fig.15.

Answer:

This is now addressed in SI Section B.3.1, where we show how RMSE on PDBBind varies with both number of features and number and estimators.

SI: Table 6; R-square values < -1 ?

Answer:

While rare, R-squared values can be < -1, which indicates a very poor model and a catastrophically bad fit. R-squared is defined as:

$$R^2 = 1 - \frac{SS_{\text{res}}}{SS_{\text{tot}}}$$

where the sum of squared residuals $SS_{\text{res}} = \sum_i (y_i - f_i)^2 = \sum_i e_i^2$, sum of squares $SS_{\text{tot}} = \sum_i (y_i - \bar{y})^2$, and mean of observed data $\bar{y} = \frac{1}{n}\sum_{i=1}^{n} y_i$. A simple example of observations and predictions with $R^2 < -1$ from the documentation for R^2 from scikit-learn, a well-established machine learning software suite [6]:

y_i	f_i
1	3
2	2
3	1

This dataset has $SS_{\text{res}} = 8$, $SS_{\text{tot}} = 2$, and therefore $R^2 = -3$. See Wikipedia page for coefficient of determination
(https://en.wikipedia.org/wiki/Coefficient_of_determination) and scikit-learn documentation for R^2(https://scikit-learn.org/stable/modules/generated/sklearn.metrics.r2_score.html).

Please check the order of references, figures, and tables appearing in the main text.

Answer:

Done. The references are now in the order they appear. The references also now follow the official PLOS format.

Reference in section heading: 4.2 The TNet-BP algorithm [13]; is not required.

Answer:

This is now fixed.

Reviewer 3

> The authors descried a topology-based approach, PATH, in structure based drug affinity prediction. They claimed that, in contrast to the more popular deep learning or physics-based approaches, PATH is interpretable and can avoid over fitting and adaptable to new targets, and it runs faster than existing topology based approach too. I must admit that this is not an area that I am familiar, although I do dabble in the drug design area. I do not feel qualified to critique this work.

Answer:

We thank the reviewer for the interest and positive evaluation of our work.

> I do have the following comments.
>
> First of all, is 120+ references necessary? and the numbering doesn't follow the order they appear in the paper, which makes me think some of these texts may be taken from a thesis.

Answer:

We fixed the ordering of the references. The references are now in the order they appear.

> Deep learning-based approach does suffer from lack of interpretability but there are ways to remedy this to identify residues or chemical groups that are important in binding.

Answer:

Yes, this is a good point. There are ways to examine the contributing factors to the prediction of a deep learning or black box model, and almost all recent algorithms on protein-ligand binding affinity prediction incorporate explainability in some form. However, meta-reviews in machine learning find that explanations of black boxes can be unreliable or misleading [7]. Therefore, a simple algorithm like PATH provides a stronger interpretability than deep learning. We added an extended discussion on this point in our discussion section.

> I felt the Results section is a little thin, as they only tested on one dataset and provided one comparison figure (Fig 4), and one case study HIV-1 protease.

Answer:

We believe the reviewer is looking at a much earlier version of our manuscript on bioRxiv or for RECOMB. The R0 manuscript (MS) which the reviewer was sent, (and also the revised R1 MS as well) includes tests on three data sets (not one), and it includes three case studies (Figures 6, 7, 9).

References

[1] Mark A Hallen et al. "OSPREY 3.0: open-source protein redesign for you, with powerful new features". In: *Journal of computational chemistry* 39.30 (2018), pp. 2494–2507.

[2] Adegoke Ojewole et al. "OSPREY predicts resistance mutations using positive and negative computational protein design". In: *Computational Protein Design* (2017), pp. 291–306.

[3] Kelly Huynh et al. "Discovery, characterization, and redesign of potent antimicrobial thanatin orthologs from Chinavia ubica and Murgantia histrionica targeting E. coli LptA". In: *Journal of Structural Biology: X* 8 (2023), p. 100091. ISSN: 2590-1524. DOI: https://doi.org/10.1016/j.yjsbx.2023.100091. URL: https://www.sciencedirect.com/science/article/pii/S2590152423000077.

[4] Graham T Holt et al. "Improved HIV-1 neutralization breadth and potency of V2-apex antibodies by in silico design". In: *Cell reports* 42.7 (2023).

[5] John D Westbrook et al. "The chemical component dictionary: complete descriptions of constituent molecules in experimentally determined 3D macromolecules in the Protein Data Bank". In: *Bioinformatics* 31.8 (2015), pp. 1274–1278.

[6] F. Pedregosa et al. "Scikit-learn: Machine Learning in Python". In: *Journal of Machine Learning Research* 12 (2011), pp. 2825–2830.

[7] Cynthia Rudin. "Stop explaining black box machine learning models for high stakes decisions and use interpretable models instead". In: *Nature machine intelligence* 1.5 (2019), pp. 206–215.
